# Huyan-I formula attenuates renal senescence and fibrosis by inhibiting STAT3/NF-κB/NLRP3-driven SASP

**DOI:** 10.1007/s11418-026-02046-1

**Published:** 2026-06-22

**Authors:** Kaizhi Wen, Xiaofan Yin, Lingling Sun, Kena Yu, Liyan Huang, Qing Song, Buhui Liu, Xinyuan Cui, Yue Tu, Weiming He

**Affiliations:** 1 First Clinic Medical School, Nanjing University of Chinese Medicine, Nanjing, People’s Republic of China; 2 Renal Division, Affiliated Hospital of Nanjing University of Chinese Medicine, Jiangsu Province Hospital of Chinese Medicine, No.155 Hanzhong Road, Qinhuai District, Nanjing, Jiangsu Province People’s Republic of China; 3 Department of Traditional Chinese Medicine Health Preservation, Acupuncture, Moxibustion and Massage College, Health Preservation and Rehabilitation College, Nanjing University of Chinese Medicine, Nanjing, 210023 People’s Republic of China; 4 Department of Human Anatomy, Xuzhou Medical University, Xuzhou, 221004 People’s Republic of China; 5 School of Medicine, Nanjing University of Chinese Medicine, Nanjing, People’s Republic of China

**Keywords:** HY-I Formula, Kidney aging, SASP, Network pharmacology, STAT3/NFκ-B/NLRP3 signaling pathway

## Abstract

The Huyan-I formula (HY-I), a patented six‑herb traditional Chinese medicine used clinically at Jiangsu Provincial Hospital of TCM for early‑stage chronic kidney disease (CKD), has demonstrated therapeutic efficacy; however, its molecular mechanisms remain unclear. Given the central role of cellular senescence and fibrosis in CKD progression, particularly through the senescence‑associated secretory phenotype (SASP), this study investigated whether HY-I ameliorates renal senescence and fibrosis by modulating SASP‑related signaling. By UPLC‑Q‑TOF–MS analysis, 211 components were identified in HY‑I, and 29 prototype compounds plus 19 metabolites were detected in the serum of HY‑I‑treated mice. Network pharmacology and molecular docking predictions indicated interactions with the STAT3/NF‑κB/NLRP3/SASP axis. In naturally aged mice and in D‑galactose‑induced senescent human renal tubular (HK‑2) cells treated with HY-I or resveratrol (positive control), HY-I significantly reduced senescent cell burden and senescence markers, suppressed activation of STAT3, NF‑κB, and NLRP3, downregulated SASP expression, and improved tubular injury and renal fibrosis as assessed by Western blot, immunohistochemistry, multiplex immunofluorescence, and histopathological staining (HE, Masson, PAS). These findings demonstrate that HY-I alleviates renal aging and fibrosis by inhibiting the STAT3/NF‑κB/NLRP3 pathway and attenuating the SASP, thereby providing a mechanistic basis for its clinical application in CKD.

**Graphical abstract:**

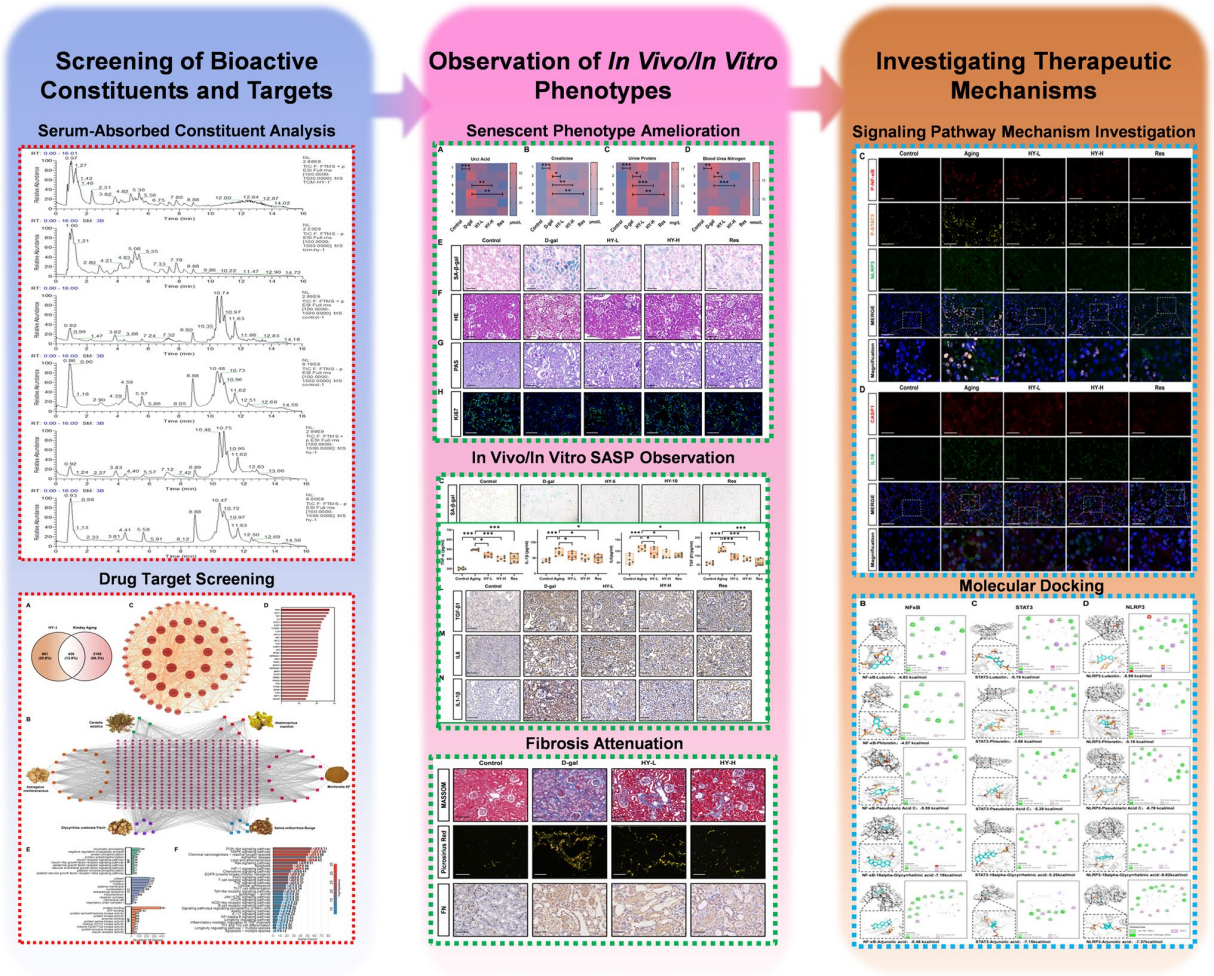

## Introduction

Chronic kidney disease (CKD) represents a major global public health challenge, affecting approximately 10% of the world’s population [1]. Beyond its association with aging and morbidity, intrinsic renal aging processes significantly increase susceptibility to CKD progression in the elderly [2].

Renal fibrosis, characterized by excessive extracellular matrix (ECM) accumulation in glomeruli, tubulointerstitium, and vasculature, is a hallmark pathological feature of CKD that ultimately leads to end-stage kidney disease (ESKD) [3]. Substantial evidence implicates cellular senescence as a key driver of renal aging and fibrosis [4]. Senescent cells accumulate in aged and diseased kidneys, secreting a senescence-associated secretory phenotype (SASP) that includes proinflammatory cytokines (IL-1β, IL-6), chemokines (IL-8, MCP-1), growth factors (TGF-β, VEGF), and proteases. These SASP components collectively promote fibrosis by inducing tubular epithelial injury, fibroblast activation, ECM degradation, and fibrous deposition.

Transcriptional regulation of SASP involves complex signaling networks. NF-κB serves as a master regulator of inflammatory responses, upregulating proinflammatory genes and chemokine-mediated immune cell recruitment [5]. NLRP3 inflammasome assembly, triggered by NF-κB, activates caspase-1 to process IL-1β/IL-18 [6]. STAT3 synergizes with NF-κB to amplify SASP transcription through IL-6 and NLRP3 upregulation [7].

Resveratrol, a natural polyphenol with documented anti-senescence and anti-inflammatory effects, inhibits NF-κB and STAT3 signaling and NLRP3 activation [8]. Notably, it rescues renal function in aging models and CKD settings [9], serving as our positive control for evaluating HY-I’s effects on senescence.

Traditional Chinese Medicine (TCM) formulas like HY-I offer multi-component, multi-target therapeutic strategies. HY-I contains six herbs: Astragali Radix, Abelmoschi Corolla, Centellae Herba, Salviae Miltiorrhizae Radix et Rhizoma, Glycyrrhizae Radix et Rhizoma, and Cordyceps mycelia. This patented formula, derived from Jiangsu nephropathy therapies, significantly reduces serum creatinine/Cystatin-C and improves eGFR in stage 2–3 CKD patients.

In this study, we identified HY-I’s bioactive serum components and predicted targets via network pharmacology. Using naturally aged mice and D-galactose-induced tubular senescence models, we investigated HY-I’s renoprotective effects and SASP-related mechanisms.

## Material and methods

### Preparation of HY-I and drug-containing serum

HY-I contains six medicinal components: *Astragalus membranaceus* (15 g), *Abelmoschus Manihot* (15 g), *Centella asiatica* (15 g), *Salvia miltiorrhiza* (10 g), *Glycyrrhiza uralensis* (4 g), and *Mortierella* sp. mycelia powder (3.75 g). All crude materials were authenticated by a medicinal plant identification specialist at Jiangsu Provincial Hospital of Traditional Chinese Medicine.

This formula was patent-certified. According to the Chinese patent (ZL202311543505.8), the individual herbal components were mixed in the specified proportions, decocted in water, and concentrated to a density of 1.25 g/ml.

Based on a standard adult dose (60 kg body weight), the equivalent mouse dose was calculated as 2.7 g/kg/day after adjustment for body surface area differences (using a standard conversion factor). Experimental groups received either this equivalent dose or a double dose. HY-I was dissolved in distilled water and administered via intragastric gavage.

For serum preparation, 4-month-old male C57BL/6 mice were administered HY-I at a dose of 2.7 g/kg daily for 7 consecutive days. Blood was collected 1.5 h after the final administration. Serum was separated and heat-inactivated at 56°C for 30 min.

### Animal study and dosing regimen

8 2-month-old and 32 18-month-old male C57BL/6 mice were purchased from Huachuang Sino Technology Co., Ltd. (Jiangsu, China). All animals were housed under specific pathogen-free conditions at Nanjing University of Chinese Medicine, with controlled temperature at 25 °C, humidity at 45%, and a 12-h light/dark cycle. The study protocol received approval from the Institutional Animal Ethics Committee of Nanjing University of Chinese Medicine under approval number 202411A022, with all procedures complying with animal research guidelines.

The eight 2-month-old mice served as the young control group. 18-month-old mice were randomly divided into four experimental groups:

Aging group: no treatment;

HY-I low-dose group receiving 2.7 g/kg/d HY-I;

HY-I high-dose group receiving 5.4 g/kg/d HY-I.

Resveratrol group receiving 40 mg/kg resveratrol (B20044, Orileaf, Shanghai).

All drugs were administered once daily via intragastric gavage for a period of two months. At the end of the intervention period, mice were anesthetized with 3% isoflurane for blood collection and then humanely euthanized by CO₂ inhalation. Serum, urine, and kidney tissue samples were collected from the mice.

### Identification of HY-I by UPLC-Q-TOF–MS

#### UPLC method for qualitative analysis

Thermo-Obritrap-QE-MS was used for quality control and chemical component identification of HY-I and its drug-containing serum. Qualitative analysis was performed on ACQUITY UPLC I-Class and the ACQUITY UPLC HSS T3 (100 mm × 2.1 mm, 1.8 µm) was used in the system. The PDA detector has a scanning range of 210—240 nm and is model ACQUITY UPLC. The column temperature was set to 45 ℃, the flow rate was set to 0.35 mL/min, the injection volume was 5 μL, and water containing 0.1% formic acid (A) and acetonitrile (B) was used as the mobile phase. The optimal linear gradient elution conditions were shown in Table 1.

**Table 1:** The mobile phase gradient

Time (min)	A% (0.1% aqueous formic acid)	B% (acetonitrile)
0	95	5
2	95	5
4	70	30
8	50	50
10	20	80
14	0	100
15	0	100
15.1	95	5
16	95	5

#### UPLC-MS method for qualitative analysis

The UPLC-MS/MS analysis was conducted on a Thermo-Obritrap-QE HF mass spectrometer equipped. A detection mode was ESI-Negative and Positive ion mode, and the mass spectrometer parameters were set as Table 2.

**Table 2:** Mass parameters

MS parameter	ESI-negative	ESI-positive
Spray voltage (V)	3800	−3200
Capillary temperature (°C)	320	320
Aux gas heater temperature (℃)	350	350
Sheath gas flow rate (Arb)	35	35
Aux gas flow rate (Arb)	8	8
S-lens RF level	50	50
Mass range (m/z)	100–1500	100–1500
Full ms resolution	60,000	60,000
MS/MS resolution	15,000	15,000
NCE/stepped NCE	10, 20, 40	10, 20, 40

#### Data processing

The UHPLC-MS/MS mode was applied with an Orbitrap resolution of 60,000 for full-MS and 15,000 for dd-MS2. The raw data was analyzed by Xcalibur3.0 software. Prior to pattern recognition, the raw data were processed using the metabolomics software Progenesis QI v3.0 (Nonlinear Dynamics, Newcastle, UK) for baseline filtering, peak identification, integration, retention time correction, peak alignment, and normalization. Compound identification was performed based on accurate mass, MS/MS fragments, and isotopic distribution patterns, with qualitative analysis conducted using the TCM database. The Traditional Chinese Medicine (TCM) database is a specialized repository meticulously developed for plant specimens. It encompasses comprehensive information on more than 5,000 reference standards of TCM components. These reference standards were procured from esteemed suppliers, including Chengdu Le Meitian Pharmaceutical Technology Co., Ltd. and Shanghai Yuanye Biotechnology Co., Ltd.

Compounds are initially screened based on a mass deviation not exceeding 5 ppm and a database match score higher than 50%. Finally, compounds are verified through a comparison of the parent ions and secondary fragment ions in the raw data with those reported in the literature.

### Cell culture and drug treatment

HK-2 cells were cultured in DMEM/F12 medium (L310KJ, Basal Media) supplemented with 10% fetal bovine serum (FBS, catalog 10,099—141C, Gibco) at 37 °C under 5% CO₂.

Cells were treated with varying concentrations of D-galactose (catalog D8310, Solarbio), HY-I-containing serum, or 10 μM resveratrol (catalog B20044, Orileaf) as the positive control group. Cell viability was assessed using CCK-8 Kit (BMU106-CN, Abbkine).

### Western blot

Cellular and renal homogenates were lysed using RIPA buffer. Proteins were separated by electrophoresis on 4–12% SDS-PAGE gels and transferred to PVDF membranes (MIPVH0010, Millipore, USA). Membranes were incubated with appropriate primary and secondary antibodies, followed by detection with ECL reagent (P90720, Millipore USA) and visualization using a gel imaging system (LAS 4000, FUJIFILM, Japan).

For membrane reprobing, PVDF membranes were incubated with western blot stripping buffer (WB6500, New Cell & Molecular Biotech, Suzhou, China) at room temperature for 20 min. After PBST washing, membranes were re-incubated with antibodies and re-detected as described above. The list of antibodies is provided in Table 6.

### Renal histopathological staining

Paraffin-embedded mouse renal sections underwent xylene deparaffinization and ethanol gradient rehydration, followed by staining with H&E (G1120,Solarbio,China), PAS (G1280,Solarbio,China), and Masson’s trichrome (G1340, Solarbio,China) according to the manufacturer’s protocol for fibrosis assessment.

### Immunofluorescence and immunohistochemistry

For IHC, antigen retrieval was performed in citrate or EDTA buffer. Endogenous peroxidase was quenched with H₂O₂, followed by BSA blocking. Primary antibodies were applied at 4°C overnight. After PBS washing, secondary antibodies were incubated for 1 h at room temperature. Signal detection used DAB.

For IF, tissues fixed in 4% PFA were permeabilized and subjected to multiplex staining using TSA Kit (Aifang Biological,Hunan,China). Nuclei were counterstained with DAPI. Images were acquired using a fluorescence microscope (Axio Vert A1, ZEISS).

### Renal function assessment

Mouse serum and urine samples were centrifuged and analyzed using commercial kits (C035—2—1, C012—2—1, C013—2—1, C011—2—1; Jiancheng, Nanjing, China) for creatinine, uric acid, blood urea nitrogen, and urinary protein according to manufacturer’s instructions.

### ELISA assays

SASP-related factors were quantified using ELISA kits: IL-6 (AF2163-A), TGFβ1 (AF2135-A), TNFα (AF2132-A), IL-1β (AF2040-A) from Aifang Biological, MMP-9(E-EL-M3052), GDF15(E-EL-M0604) from Elabscience Biological. Renal homogenates were incubated with detection reagents per protocol. After TMB substrate incubation at 37 °C for 15 min, reactions were stopped and absorbance measured at 450 nm.

### SA-β-gal activity staining

Cryosections (8 μm) and cultured cells were stained using SA-β-gal staining kit (C0602, Beyotime, Shanghai, China) with nuclear fast red counterstain (G1320,Solarbio,Beijing,China) for tissue sections.

### Establishment of a compound-target network

#### Collection of the targets

Targets of ingredients: The Canonical SMILES format of the HY-I ingredients was downlorded from PubChem (https://pubchem.ncbi.nlm.nih.gov/). The potential targets for these ingredients were searched through the Swiss Target Prediction (http://swisstargetprediction.ch/).

Targets of kidney aging: kidney aging targets were identified using several databases, Gene Cards (https://www.genecards.org/), OMIM (https://omim.org/), Drug Bank (https://go.drugbank.com/), taking “kidney aging” as the search term. The targets gathered from these databases were subsequently merged, and any duplicate entries were eliminated. UniProt (https://www.uniprot.org) was employed to convert the targets into standardized Gene Symbols. Finally, the online Venn diagram website, Venny 2.1 (https://bioinfogp.cnb.csic.es/tools/ venny/index.html), was utilized to visually represent a Venn diagram that described the intersection of HY-I and kidney aging. A compound-target network, based on HY-I compounds and their potential targets, was visualized using Cytoscape version 3.7.1.

#### Protein–protein interaction (PPI) network construction

The cross-target was uploaded to the STRING (https://www.string-db.org/) database, and the species “Homo sapiens” was selected. A protein–protein interaction (PPI) network was constructed with a high confidence level > 0.4. Subsequently, the network topology was analyzed and visualized with the aid of Cytoscape version 3.7.1.

#### GO and KEGG pathway enrichment

The intersected targets were entered into David (https://davidbioinformatics.nih.gov/) for biofunctional enrichment and pathway enrichment analysis. In accordance with the research topic, the top 30 results were selected based on their P-values to examine their main pathways and biological processes. Visualizations were generated using R software (v4.2.3).

### Molecular docking

Molecular docking studies were performed to investigate the binding interactions between the main constituents of the HY-I formulation and disease-related targets. The three-dimensional structures of these compounds were retrieved from PubChem (https://www.ncbi.nlm.nih.gov/pccompound), and the corresponding protein structures of the targets were obtained from the Protein Data Bank (https://www.rcsb.org). Molecular docking was performed using AutoDock 4.2. After docking, the conformation exhibiting the lowest binding energy and the highest frequency of recurrent binding poses was selected as the optimal result. The selected complex was subsequently imported into PyMOL 3.1 and Discovery Studio 2021 for visualization and further structural analysis. The PDB ID corresponding to each docking target is listed in Table 7.

Molecular Dynamics MD simulations were carried out with GROMACS 2022.3. The CHARMM36 force field described the proteins, and ligand parameters were derived from the CGenFF server. Each protein–ligand complex was centered in a 1.0‑nm cubic box filled with TIP3P water. NaCl was added to 0.15 M, and counterions neutralized the system.

Initial energy minimization used the steepest descent method (5000 steps, 0.01‑nm step size). The system was then equilibrated for 100 ps under NVT at 310 K (V‑rescale thermostat), followed by 100 ps under NPT at 1 bar (Parrinello–Rahman barostat). Bond lengths were constrained via LINCS. A 1.2‑nm cutoff was applied for non‑bonded interactions, and PME handled long‑range electrostatics.

Production runs lasted 100 ns with a 1 fs time step, saving coordinates every 10 ps. Each complex was simulated in triplicate. The four simulated complexes were: STAT3–18α‑glycyrrhetinic acid, NLRP3–18α‑glycyrrhetinic acid, NLRP3–arjunolic acid, and NF‑κB–pseudolaric acid C.

### Statistical analysis

Statistical analyses were performed using GraphPad Prism 9.0. All values were expressed as mean ± standard errors of the means (SEMs). All the data were from at least three independent experiments. Differences among multiple groups were determined by one-way analysis of variance (ANOVA) followed by Tukey’s post-hoc test. Values were considered significantly different at *p* < 0.05.

## Results

### Major and serum-absorbed constituents of HY-I

**Fig. 1:**
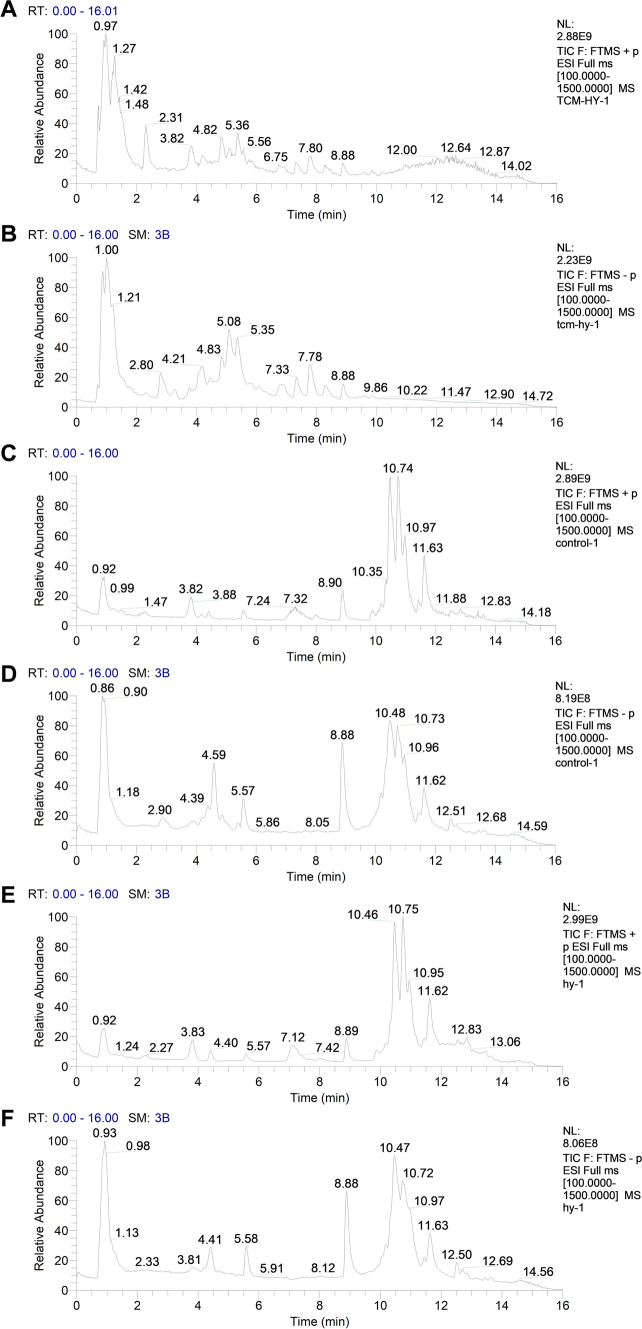
Major and serum-absorbed constituents of HY-I. Typical total ion chromatograms (TIC) in both positive and negative ion modes. TIC chromatograms of HY-I in positive (**A**) and negative (**B**) ion modes. TIC chromatograms of Blank serum in positive (**C**) and negative (**D**) ion modes. TIC chromatograms of HY-I medicated serum in positive (**E**) and negative (**F**) ion modes

The chemical constituents of HY-I (Fig. 1A, B and Table 3) and HY-I containing serum (Fig. 1E, F and Tables 4, 5) were analyzed using ultra-high performance liquid chromatography coupled with quadrupole-Orbitrap mass spectrometry (Thermo Orbitrap QE). Based on the multistage mass spectrometry data of the samples and by referencing the natural product high-resolution mass spectrometry database, a total of 211 compounds were identified from HY-I, including 29 prototype components and 19 metabolites detected in serum (see Tables 6, 7). The following criteria were used to define blood‑absorbed components. A component was considered a blood‑absorbed prototype compound if it was present in either blank serum or drug‑containing serum, with a peak area ratio relative to blank serum ≥ FC (fold change threshold), or if the peak was undetectable in blank serum. A component detected in drug‑containing serum and verified by mass spectrometry as a metabolite of a prototype compound was designated as a blood‑absorbed metabolite.

**Table 3:** Identification of chemical constituents of HY-I based on Thermo-Obritrap-QE

No.	English name	RT	Formula	Calc. MW	Adducts	Error (ppm)	Theoretical Mass (m/z)	Experimental mass (m/z)	MS^2^ (m/z)	Score	Source
1	N6,N6,N6-Trimethyl-L-lysine	0.77	C_9_H_20_N_2_O_2_	188.1519	[M + H]^+^	−1.55	189.1598	189.1595	60.0813, 70.0656, 74.0718, 84.0812, 116.0707, 126.0916, 130.0861, 189.1346, 189.1593	53.7	/
2	Galactose 1-phosphate	0.77	C_6_H_13_O_9_P	260.0292	[M − H]^−^	−1.05	259.0224	259.0222	78.959, 96.9694, 259.0216	51.6	/
3	L-Histidine	0.79	C_6_H_9_N_3_O_2_	155.0689	[M − H]^−^	−1.01	154.0622	154.0620	93.0458, 94.9245, 96.9599, 110.0723, 137.0352, 154.0618	57.2	*Glycyrrhiza uralensi*s Fisch、Cordycepssinensis、Astragalus membranaceus、Abelmoschus manihot
4	Maltopentaose	0.79	C_30_H_52_O_26_	828.2741	[M + FA-H]^−^	0.17	873.2729	873.2730	89.0241, 96.9599, 179.0554, 208.554, 383.1181, 827.2669	48.2	Astragalus membranaceus
5	D-mannitol	0.81	C_6_H_14_O_6_	182.0785	[M − H]^−^	−1.30	181.0718	181.0715	59.0137, 71.0138, 73.0294, 85.0294, 89.0244, 101.0243, 119.0348, 163.0609, 181.0715	57.3	Cordycepssinensis
6	L-Arginine	0.81	C_6_H_14_N_4_O_2_	174.1111	[M − H]^−^	−1.20	173.1044	173.1042	131.0824, 156.0775, 173.1052	57.7	Astragalus membranaceus, Cordycepssinensis
7	Stachyose	0.81	C_24_H_42_O_21_	666.2213	[M − H]^−^	0.20	711.2201	711.2202	89.0244, 179.056, 383.1195, 665.2175	56.7	Glycyrrhiza uralensis、Astragalus membranaceus
8	L-Asparagine	0.81	C_4_H_8_N_2_O_3_	132.0529	[M − H]^−^	−1.39	131.0462	131.0460	73.0293, 85.0294, 88.0400, 95.0251, 103.2722, 113.0239, 113.0354, 114.0196, 115.0033, 131.0452	55.8	*Glycyrrhiza uralensi*s Fisch、Astragalus membranaceus
9	D-Tagatose	0.83	C_6_H_12_O_6_	180.0628	[M − H]^−^	−0.99	179.0561	179.0559	59.0137, 71.0137, 85.0293, 89.0244, 101.0243, 113.0242, 119.0349, 121.0437, 161.0455, 179.0551	55.1	Cordycepssinensis、Astragalus membranaceus
10	Turanose	0.85	C_12_H_22_O_11_	342.1157	[M − H]^−^	−2.08	387.1145	387.1137	89.0251, 119.034, 179.0566, 341.1084	58.2	Astragalus membranaceus、Salvia miltiorrhiza Bunge、Cordycepssinensis
11	Manninotriose	0.85	C_18_H_32_O_16_	504.1685	[M − H]^−^	−1.30	549.1673	549.1666	71.0138, 89.0244, 179.0558, 221.0664, 503.1614, 549.1666	57.8	Astragalus membranaceus、Salvia miltiorrhiza Bunge
12	L-Glutamic acid	0.87	C_5_H_9_NO_4_	147.0526	[M − H]^−^	−1.23	146.0459	146.0457	93.8274, 95.9757, 99.4594, 102.0558, 125.5103, 126.4884, 128.0351, 140.2054, 142.9211, 146.0453	55	*Glycyrrhiza uralensi*s Fisch、Cordycepssinensis、Astragalus membranaceus
13	D-altrofurano-heptulose-3	0.88	C_7_H_14_O_7_	210.0734	[M – 2OH – H]^–^	−1.46	191.0561	191.0558	85.0295, 87.0087, 111.0087, 173.0089, 191.0211, 191.0559	55.6	/
14	Gluconic acid	0.88	C_6_H_12_O_7_	196.0578	[M − H]^−^	−1.19	195.0510	195.0508	75.0087, 87.0087, 99.0087, 129.0192, 159.0296, 177.0403, 195.0507	58.5	Astragalus membranaceus、Glycyrrhiza uralensis、Cordycepssinensis
15	Sucrose	0.88	C_12_H_22_O_11_	342.1157	[M + Na]^+^	−3.44	365.1055	365.1043	185.0416, 203.052, 365.1039	56.1	Glycyrrhiza uralensis、Astragalus membranaceus
16	Malic acid	0.94	C_4_H_6_O_5_	134.0210	[M − H]^−^	−1.41	133.0142	133.0141	71.0138, 72.9929, 115.0036, 133.014	52.6	Glycyrrhiza uralensis、Astragalus membranaceus、Abelmoschus manihot
17	L-Threonine	0.95	C_4_H_9_NO_3_	119.0577	[M + H]^+^	0.32	120.0655	120.0656	56.05, 74.0605, 102.0548, 120.0557, 120.0651	54.4	Glycyrrhiza uralensis、Astragalus membranaceus、Abelmoschus manihot、Salvia miltiorrhiza Bunge
18	DL-Proline	0.95	C_5_H_9_NO_2_	115.0628	[M + H]^+^	1.42	116.0706	116.0708	70.0656, 116.0706	67.4	Glycyrrhiza uralensis、Astragalus membranaceus、Abelmoschus manihot、Astragalus membranaceus
19	DL-Valine	0.95	C_5_H_11_NO_2_	117.0784	[M + H]^+^	0.75	118.0863	118.0863	58.0657, 59.0735, 72.0813, 118.0863	53.2	Cordycepssinensis
20	Cytosine	0.99	C_4_H_5_N_3_O	111.0427	[M + H]^+^	2.03	112.0505	112.0508	69.0453, 70.0656, 95.0243, 112.0506	53.6	*Glycyrrhiza uralensi*s Fisch、Cordycepssinensis
21	Cytidine	0.99	C_9_H_13_N_3_O_5_	243.0850	[M + H]^+^	−2.46	244.0928	244.0922	70.0656, 112.0506, 244.0784	52.4	Cordycepssinensis
22	Valylalanine	1.01	C_8_H_16_N_2_O_3_	188.1155	[M + H]^+^	−2.01	189.1234	189.1230	84.0813, 118.0863, 126.0913, 129.0551, 130.0499, 130.0862, 143.1177, 144.1017, 172.0961, 189.1243	53.1	/
23	Citric acid	1.03	C_6_H_8_O_7_	192.0265	[M − H]^−^	−0.90	191.0197	191.0196	85.0294, 87.0087, 111.0086, 129.019, 191.0193	60.2	Astragalus membranaceus、*Glycyrrhiza uralensi*s Fisch、Salvia miltiorrhiza Bunge、Abelmoschus manihot
24	Pinitol	1.05	C_7_H_14_O_6_	194.0785	[M + FA-H]^−^	−1.19	239.0773	239.0770	193.0703, 195.0288, 195.1377, 201.9223, 238.8911, 239.0168, 239.0230, 239.0551, 239.0626, 239.0770	53.9	Astragalus membranaceus
25	L-Pipecolic acid	1.05	C_6_H_11_NO_2_	129.0784	[M + H]^+^	−0.49	130.0863	130.0862	70.0656, 84.045, 84.0812, 130.0497, 130.086	51.8	/
26	DL-Pyroglutamic acid	1.11	C_5_H_7_NO_3_	129.0420	[M − H]^−^	−1.36	257.0779	257.0776	128.0352, 239.0666, 257.0766	53	Glycyrrhiza uralensis、Cordycepssinensis、Astragalus membranaceus
27	Cyclic AMP	1.16	C_10_H_12_N_5_O_6_P	329.0520	[M − H]^−^	−1.93	328.0452	328.0446	134.047, 328.0442	53.8	Glycyrrhiza uralensis、Cordycepssinensis、Astragalus membranaceus
28	5′-GMP	1.16	C_10_H_14_N_5_O_8_P	363.0575	[M − H]^−^	−1.49	362.0507	362.0502	78.9589, 150.0417, 211.0005, 362.05	56.8	Cordycepssinensis
29	Cyclic GMP	1.18	C_10_H_12_N_5_O_7_P	345.0469	[M − H]^−^	−2.41	344.0402	344.0393	133.0153, 150.0419, 344.0389	58.2	Cordycepssinensis
30	Uridine	1.22	C_9_H_12_N_2_O_6_	244.0690	[M − H]^−^	−1.42	243.0623	243.0619	82.0297, 110.0247, 111.0195, 115.0034, 128.0352, 140.035, 152.0353, 153.0303, 200.0563, 243.0621	73.2	Glycyrrhiza uralensis、Cordycepssinensis、Astragalus membranaceus、Abelmoschus manihot
31	N-Acetyl-L-glutamic acid	1.26	C_7_H_11_NO_5_	189.0632	[M − H]^−^	−0.23	188.0564	188.0564	100.0767, 102.0559, 116.0714, 126.0275, 126.0558, 128.0351, 144.0664, 146.0455, 170.0456, 188.0563	58.6	Cordycepssinensis、Astragalus membranaceus
32	Adenosine	1.27	C_10_H_13_N_5_O_4_	267.0962	[M + H]^+^	−2.75	268.1040	268.1033	136.0616, 268.1034	61.1	Astragalus membranaceus、Salvia miltiorrhiza Bunge、Cordycepssinensis
33	L-2-Hydroxyglutaric acid	1.36	C_5_H_8_O_5_	148.0366	[M − H]^−^	−1.07	147.0299	147.0297	75.0085, 85.0292, 87.0088, 103.0393, 129.0195, 147.0296	53.5	/
34	Cordycepin	1.4	C_10_H_13_N_5_O_3_	251.1013	[M + H]^+^	−2.48	252.1091	252.1085	136.0615, 252.1076	65	Cordycepssinensis
35	Methylmalonic acid	1.4	C_4_H_6_O_4_	118.0261	[M − H]^−^	−0.66	117.0193	117.0193	73.0295, 99.0087, 116.9284, 117.0192	57.3	/
36	Guanosine	1.46	C_10_H_13_N_5_O_5_	283.0911	[M − H]^−^	−1.31	282.0844	282.0840	133.015, 150.042, 282.0836	55.9	Cordycepssinensis、Astragalus membranaceus、Salvia miltiorrhiza Bunge
37	L-Leucine	1.51	C_6_H_13_NO_2_	131.0941	[M + H]^+^	−0.75	132.1019	132.1018	86.097	68	Glycyrrhiza uralensis、Cordycepssinensis、Astragalus membranaceus、Abelmoschus manihot、Salvia miltiorrhiza Bunge
38	2-Methylcitric acid	1.55	C_7_H_10_O_7_	206.0421	[M − H]^−^	−0.69	205.0354	205.0352	71.0501, 87.0087, 99.045, 101.0243, 111.0086, 125.0242, 187.0244, 205.035	60.7	/
39	Scandoside	1.67	C_16_H_22_O_11_	390.1157	[M 2OH – H]^–^	−1.22	371.0983	371.0979	135.045, 173.0452, 179.0348, 191.0559, 197.0456, 209.03, 353.0881, 371.0988	61.3	/
40	Gallic acid	1.8	C_7_H_6_O_5_	170.0210	[M − H]^−^	−0.44	169.0142	169.0142	125.0243, 169.014	64.7	Glycyrrhiza uralensis、Cordycepssinensis、Astragalus membranaceus、Abelmoschus manihot、Salvia miltiorrhiza Bunge
41	2′-O-Methyladenosine	1.94	C_11_H_15_N_5_O_4_	281.1119	[M + H]^+^	−2.16	282.1197	282.1191	136.0615, 282.1182	51.5	Cordycepssinensis
42	Benzaldehyde	2.28	C_7_H_6_O	106.0413	[M + H]^+^	2.87	107.0491	107.0494	53.0393, 79.0547, 91.0545, 95.0497, 107.049	52.3	Cordycepssinensis
43	L-Phenylalanine	2.32	C_9_H_11_NO_2_	165.0784	[M − H]^−^	−1.46	164.0717	164.0715	72.0098, 96.9601, 96.9691, 115.9205, 116.9283, 134.8943, 147.0449, 164.0338, 164.0712	57.2	Glycyrrhiza uralensis、Cordycepssinensis、Astragalus membranaceus、Salvia miltiorrhiza Bunge
44	N-(1-Deoxy-D-fructos-1—yl)-L-phenylalanine	2.36	C_15_H_21_NO_7_	327.1313	[M + H]^+^	−3.37	328.1391	328.1380	120.0808, 132.0806, 166.0859, 264.1223, 292.1171, 310.1275, 328.1378	67.9	Astragalus membranaceus
45	Ser-Leu	2.6	C_9_H_18_N_2_O_4_	218.1261	[M + H]^+^	−1.47	219.1339	219.1336	60.0449, 86.0968, 132.1017, 173.128, 201.122, 203.1425, 205.1583, 219.1116, 219.1333	54.5	/
46	Glycylleucine	2.6	C_8_H_16_N_2_O_3_	188.1155	[M + H]^+^	−0.38	189.1234	189.1233	75.0558, 84.0811, 86.0968, 98.0599, 118.0862, 129.0542, 132.1017, 143.1175, 144.0655, 189.1117	59.3	/
47	Ethylmalonic acid	2.63	C_5_H_8_O_4_	132.0417	[M − H]^−^	−1.22	131.0350	131.0348	85.0297, 87.0451, 113.0243, 131.0348	53.3	/
48	2,4,6-Trihydroxybenzoic acid	2.75	C_7_H_6_O_5_	170.0210	[M − H]^−^	-1.17	169.0142	169.0140	125.0244, 151.0034, 169.014	58.3	/
49	Glycylisoleucine	2.95	C_8_H_16_N_2_O_3_	188.1155	[M + H]^+^	−0.59	189.1234	189.1233	86.0968, 132.1017, 143.1175	57.5	/
50	Vanillic acid 4-beta-D-glucopyranoside	3.1	C_14_H_18_O_9_	330.0945	[M − H]^−^	−0.93	329.0878	329.0875	108.0216, 123.0451, 152.0111, 167.0348, 329.0875	50.8	Salvia miltiorrhiza Bunge
51	Aspartyl-Leucine	3.15	C_10_H_18_N_2_O_5_	246.1210	[M + H]^+^	−2.03	247.1289	247.1283	141.1020, 155.1175, 166.0860, 183.1124, 187.1068, 201.1228, 212.0916, 229.1177, 247.1084, 247.1293	58.2	/
52	Glutamylleucine	3.2	C_11_H_20_N_2_O_5_	260.1367	[M + H]^+^	−2.37	261.1445	261.1439	84.0447, 86.0969, 102.0553, 132.1019, 197.1281, 225.124, 243.1333, 261.1235	55.7	/
53	Succinyladenosine	3.2	C_14_H_17_N_5_O_8_	383.1072	[M + H]^+^	−3.23	384.1150	384.1137	162.0771, 234.0609, 252.072, 384.113	60.8	Astragalus membranaceus
54	Protocatechuic acid	3.29	C_7_H_6_O_4_	154.0261	[M − H]^−^	−0.98	153.0193	153.0192	109.0293, 153.0188	60.6	Glycyrrhiza uralensis、Astragalus membranaceus、Abelmoschus manihot、Salvia miltiorrhiza Bunge
55	Serylphenylalanine	3.62	C_12_H_16_N_2_O_4_	252.1105	[M + H]^+^	−0.98	253.1183	253.1180	60.045, 70.0657, 120.0808, 166.0859, 197.1169, 207.1123, 235.1064, 253.1189	61.4	/
56	Caftaric acid	3.71	C_13_H_12_O_9_	312.0476	[M − H]^−^	−0.50	311.0409	311.0407	135.0445, 149.0087, 179.0346, 311.0385	55.9	Abelmoschus manihot
57	Glycyl-Phenylalanine	3.73	C_11_H_14_N_2_O_3_	222.0999	[M + H]^+^	−1.29	223.1077	223.1074	70.0656, 72.0813, 86.0969, 120.0808, 166.0859, 177.1018, 205.0969, 223.1074	61.1	/
58	Isoleucyl-Valine	3.73	C_11_H_22_N_2_O_3_	230.1625	[M + H]^+^	−1.42	231.1703	231.1700	72.0813, 86.0968, 132.1016, 185.1644, 231.17	60.1	/
59	Neochlorogenic acid	3.75	C_16_H_18_O_9_	354.0945	[M − H]^−^	−0.92	353.0878	353.0875	135.045, 179.0348, 191.0559, 353.0875	70.7	Abelmoschus manihot、Salvia miltiorrhiza Bunge
60	Alanylphenylalanine	3.81	C_12_H_16_N_2_O_3_	236.1155	[M + H]^+^	−1.69	237.1234	237.1230	84.0812, 86.0969, 120.0807, 142.0494, 166.0859, 175.1225, 191.1177, 192.0650, 237.0911, 237.1236	63.4	/
61	Hydroxyphenyllactic acid	3.85	C_9_H_10_O_4_	182.0574	[M − H]^−^	−0.77	181.0506	181.0505	71.0138, 72.9930, 89.0244, 101.0243, 119.0501, 135.0450, 137.0241, 163.0399, 181.0147, 181.0506	59.4	Cordycepssinensis、、Salvia miltiorrhiza Bunge
62	10-Hydroxymajoroside	3.93	C_17_H_24_O_11_	404.1313	[2 M-H]^−^	0.66	807.2564	807.2570	807.2570	41.3	Abelmoschus manihot
63	Aspartylphenylalanine	3.94	C_13_H_16_N_2_O_5_	280.1054	[M + H]^+^	−1.19	281.1132	281.1129	86.0967, 88.0396, 120.0807, 136.0614, 162.0408, 166.0859, 235.1072, 246.0754, 263.1020, 281.1120	57.4	/
64	N-Acetyl-L-tyrosine	3.97	C_11_H_13_NO_4_	223.0839	[M − H]^−^	−0.91	222.0772	222.0770	58.0295, 107.0495, 163.0395, 178.0861, 179.0352, 180.0664, 205.0344, 222.077	56.9	/
65	4-Carboxycarbostyril	4.07	C_10_H_7_NO_3_	189.0420	[M + H]^+^	−0.73	190.0499	190.0497	144.1017, 162.0545, 190.0495	59.5	/
66	3,4-Dihydroxybenzaldehyde	4.09	C_7_H_6_O_3_	138.0311	[M + H]^+^	0.20	139.0390	139.0390	65.039, 93.0336, 111.0442, 121.0282, 139.0387	57.1	Astragalus membranaceus
67	Cryptochlorogenic acid	4.17	C_16_H_18_O_9_	354.0945	[M − H]^−^	−1.49	353.0878	353.0873	135.045, 173.0452, 179.0348, 191.0559, 353.0874	65.6	Salvia miltiorrhiza Bunge、Abelmoschus manihot
68	Cis-Ferulic acid 4-O-beta-D-glucopyranoside	4.34	C_16_H_20_O_9_	356.1102	[M − H]^−^	−0.93	355.1035	355.1031	149.0605, 173.0450, 175.0513, 178.0267, 191.0195, 191.0556, 193.0496, 209.0300, 224.0675, 355.0953	55.9	/
69	Myricetin 3—o-beta-d-xylopyranosyl(1—2)-beta-D-glucopyranoside	4.42	C_26_H_28_O_17_	612.1321	[M − H]^−^	−0.32	611.1254	611.1252	271.0248, 316.022, 611.125	75.2	/
70	Cynarin	4.46	C_25_H_24_O_12_	516.1262	[M − H]^−^	−1.13	515.1195	515.1189	135.045, 161.0244, 179.0348, 191.0559, 335.0768, 353.0874, 515.119	69.1	/
71	3-O-caffeoylshikimic acid	4.48	C_16_H_16_O_8_	336.0840	[M − H]^−^	−0.61	335.0772	335.0770	93.0342, 111.0438, 135.0449, 155.0347, 161.0241, 173.045, 179.0347, 335.0775	63	/
72	3-O-p-coumaroylquinic acid	4.48	C_16_H_18_O_8_	338.0996	[M − H]^−^	−0.88	337.0929	337.0926	93.0348, 163.04, 173.0453, 191.0559, 337.0925	71.1	/
73	Luteolin-3′,7—di-glucuronide	4.5	C_27_H_26_O_18_	638.1114	[M − H]^−^	0.36	637.1046	637.1049	285.0397, 317.0295, 351.056, 461.0722, 479.0787, 637.1017	52.9	/
74	Salvianic acid c	4.5	C_18_H_18_O_9_	378.0945	[M − H]^−^	−0.93	377.0878	377.0875	72.993, 135.0451, 137.0243, 161.0241, 178.0508, 179.0345, 197.0454, 359.0769, 377.0931	65.7	Salvia miltiorrhiza Bunge
75	Schaftoside	4.52	C_26_H_28_O_14_	564.1474	[M + H]^+^	−1.07	565.1552	565.1546	451.1017, 457.1102, 469.1122, 481.1120, 493.1120, 499.1225, 511.1221, 529.1326, 547.1434, 565.1542	63.1	/
76	Quercetin 3-gentiobioside	4.54	C_27_H_30_O_17_	626.1478	[M + H]^+^	−1.84	627.1556	627.1544	85.0288, 303.049, 317.0651, 319.043, 465.1018, 481.0961	63.2	/
77	5,7-Dihydroxy-4-oxo-4H-chromene-2-carboxylic acid	4.56	C_10_H_6_O_6_	222.0159	[M − H]^−^	−0.50	221.0092	221.0091	133.0294, 177.0191, 177.055, 221.009	58	/
78	Quercetin 3-sambubioside	4.6	C_26_H_28_O_16_	596.1372	[M − H]^−^	0.00	595.1305	595.1305	271.0246, 300.027, 595.1301	60.4	Abelmoschus manihot
79	Doryphornine	4.62	C_11_H_11_NO_3_	205.0733	[M + FA-H]^−^	−0.98	250.0721	250.0719	88.0403, 115.0036, 132.0301, 135.0449, 250.0717	62	/
80	3-Feruloylquinic acid	4.62	C_17_H_20_O_9_	368.1102	[M − H]^−^	−1.32	367.1035	367.1030	93.0345, 134.0371, 173.0452, 191.0558, 193.0503, 367.1032	67.7	Salvia miltiorrhiza Bunge
81	(E/Z)-ferulic acid	4.62	C_10_H_10_O_4_	194.0574	[M -H_2_O + H] +	−0.35	177.0546	177.0546	76.0510, 89.0389, 117.0336, 118.0499, 145.0282, 149.0230, 149.0594, 160.0715, 163.0385, 177.0540	56.8	Astragalus membranaceus
82	Rutin	4.72	C_27_H_30_O_16_	610.1528	[M + H]^+^	0.59	611.1607	611.1610	85.0288, 303.049, 465.1019	72.3	Astragalus membranaceus、Abelmoschus manihot
83	Aloe-emodin-8-O-beta-D-glucopyranoside	4.79	C_21_H_20_O_10_	432.1051	[M − H]^−^	−1.41	431.0984	431.0978	113.0244, 139.1761, 153.0183, 175.0242, 208.4511, 208.4739, 209.3472, 255.0657, 431.0968	45.8	Astragalus membranaceus
84	Liquiritigenin-7—o-apiosyl(1—2)-glucoside	4.79	C_26_H_30_O_13_	550.1681	[M − H]^−^	−0.76	549.1614	549.1609	135.0086, 255.0661, 549.1613	61	Astragalus membranaceus
85	Taxifolin 7-O-rhamnoside	4.8	C_21_H_22_O_11_	450.1157	[M + H-H_2_O] +	−2.12	433.1129	433.1120	313.0695, 323.0909, 337.0698, 349.0700, 361.0684, 367.0800, 379.0804, 397.0909, 415.1018, 433.1120	54.2	/
86	Calycosin-7-O-beta-D-glucoside	4.82	C_22_H_22_O_10_	446.1207	[M + H]^+^	−3.29	447.1286	447.1271	285.0751, 447.1275	70.1	Astragalus membranaceus
87	Neoliquiritin	4.84	C_21_H_22_O_9_	418.1258	[M + H]^+^	−2.70	419.1337	419.1325	137.0229, 147.0437, 257.0802	69.6	Glycyrrhiza uralensis
88	Apigenin 7-O-(2G-rhamnosyl)gentiobioside	4.85	C_33_H_40_O_19_	740.2158	[M – 2OH – H]^−^	−0.42	721.1985	721.1982	353.0658, 383.0759, 457.1135, 463.0882, 721.1985	63.2	/
89	( +)-3,4′,5,7-Flaventetrol	4.86	C_15_H_14_O_5_	274.0836	[M + H-H_2_O] +	−2.00	257.0808	257.0803	137.0231, 147.0438, 257.0804	57.5	/
90	Liquiritin	4.87	C_21_H_22_O_9_	418.1258	[M − H]^−^	−1.78	417.1191	417.1184	119.0500, 135.0086, 153.0191, 175.0399, 179.0360, 197.0455, 255.0661, 373.0926, 417.0763, 417.1194	60.1	Glycyrrhiza uralensis
91	3-Coumaric acid	4.87	C_9_H_8_O_3_	164.0468	[M − H]^−^	−1.3	163.0401	163.0399	119.0501, 163.0399	57.3	/
92	Suberic acid	4.89	C_8_H_14_O_4_	174.0887	[M − H]^−^	−1.49	173.0819	173.0817	111.0814, 129.092, 173.0818	57.8	/
93	3,4-Dimethoxybenzyl alcohol	4.96	C_9_H_12_O_3_	168.0781	[M + NH_4_]^+^	−1.43	186.1125	186.1122	83.086, 111.0808	40.4	/
94	Quercetin 3-O-malonylglucoside	4.97	C_24_H_22_O_15_	550.0953	[M − H]^−^	−0.61	549.0886	549.0883	135.0086, 153.0191, 255.0658, 271.0244, 300.0271, 505.0982	69.3	Abelmoschus manihot
95	Vanillin	4.98	C_8_H_8_O_3_	152.0468	[M + H]^+^	−1.13	153.0546	153.0544	95.0860, 97.0649, 107.0858, 109.1015, 110.0364, 111.0442, 125.0597, 134.0596, 135.1166, 153.0543	57.8	Astragalus membranaceus、*Glycyrrhiza uralensi*s Fisch、Salvia miltiorrhiza Bunge
96	Methyl protocatechuate	4.99	C_8_H_8_O_4_	168.0417	[M − H]^−^	−1.29	167.0350	167.0348	152.0114, 167.0348	69.5	/
97	Nepitrin	5.01	C_22_H_22_O_12_	478.1106	[M − H − H_2_O]^−^	−0.47	459.0933	459.0931	459.0931	42.5	/
98	1,3-Dicaffeoylquinic acid	5.01	C_25_H_24_O_12_	516.1262	2[M − H]-	−0.67	1031.2463	1031.2456	173.0453, 179.0347, 191.0559, 353.0875, 515.1187, 1031.2421	60.7	/
99	Breynioside a	5.01	C_19_H_20_O_9_	392.1102	[M – H] – 2OH	−1.45	373.0929	373.0923	72.993, 175.0398, 179.0347, 197.0453, 257.0826, 329.1031, 373.093	63.7	/
100	Isochlorogenic acid C	5.02	C_25_H_24_O_12_	516.1262	[M + H]^+^	−2.31	517.1341	517.1329	163.0386, 499.1227	59.4	Salvia miltiorrhiza Bunge、Abelmoschus manihot
101	Kaempferol 3-O-D-galactoside	5.03	C_21_H_20_O_11_	448.1000	[M − H]^−^	−1.49	447.0933	447.0926	255.0295, 284.0323, 447.0927	60.4	/
102	kaempferol-O-glucuronide	5.03	C_21_H_18_O_12_	462.0793	[M − H]^−^	−1.03	461.0725	461.0721	113.0244, 175.0245, 285.04, 461.0719	62.1	/
103	Trifolin	5.04	C_21_H_20_O_11_	448.1000	[M + H]^+^	−2.01	449.1078	449.1069	287.0544, 288.0575, 303.049	63.2	/
104	Gossypetin	5.09	C_15_H_10_O_8_	318.0370	[M − H]^−^	−4.54	317.0303	317.0288	139.0035, 166.9983, 317.0297	58.5	Abelmoschus manihot
105	Hibifolin	5.1	C_21_H_18_O_14_	494.0691	[M − H]^−^	−1.11	493.0624	493.0618	295.0602, 317.0298, 493.0532, 493.062	72.1	/
106	Astilbin	5.12	C_21_H_22_O_11_	450.1157	[M + H-H_2_O] +	−2.27	433.1129	433.1119	271.0594, 272.063, 433.1078	54.5	/
107	Salvianolic acid E	5.12	C_36_H_30_O_16_	718.1528	[M + Na]^+^	−1.93	741.1427	741.1412	70.0667, 86.0967, 345.0362, 363.0482, 381.0581, 543.0893, 561.0999, 741.1422	60.4	Salvia miltiorrhiza Bunge
108	Hydrangetin	5.12	C_10_H_8_O_4_	192.0417	[M + H]^+^	−1.23	193.0495	193.0493	81.0703, 133.0281, 133.1008, 193.0492	63.1	/
109	Salvianolic acid H	5.22	C_27_H_22_O_12_	538.1106	[M − H]^−^	−1.42	537.1038	537.1031	135.0449, 159.0444, 179.0348, 185.0242, 197.0443, 295.0608, 313.0703, 339.0503, 493.1133, 537.1030	74.4	Salvia miltiorrhiza Bunge
110	Spiraeoside	5.26	C_21_H_20_O_12_	464.0949	[M + H]^+^	−2.28	465.1028	465.1017	85.0288, 303.049, 465.1019	59.9	Abelmoschus manihot
111	Rosmarinic	5.26	C_18_H_16_O_8_	360.0840	[M − H]^−^	−1.52	359.0772	359.0767	72.993, 135.045, 161.0242, 179.0348, 197.0453, 359.0772	67.8	Astragalus membranaceus、Salvia miltiorrhiza Bunge
112	Licraside	5.28	C_26_H_30_O_13_	550.1681	[M − H]^−^	−0.82	549.1614	549.1609	119.05, 135.0086, 153.0191, 255.0661, 549.1614	66.7	Astragalus membranaceus
113	Isoliquiritin apioside	5.32	C_26_H_30_O_13_	550.1681	[M + H]^+^	−2.54	551.1759	551.1745	137.023, 147.0437, 257.0802, 303.049, 419.1327	65.3	Astragalus membranaceus
114	Salvianolic B	5.32	C_36_H_30_O_16_	718.1528	[M + NH_4_]^+^	−1.77	736.1872	736.1859	135.044, 139.0388, 163.0388, 181.0493, 277.0486, 295.059, 323.0541, 521.1069	66.4	Salvia miltiorrhiza Bunge
115	m-Methoxyphenol	5.32	C_7_H_8_O_2_	124.0519	[M + H – H_2_O]^+^	1.67	107.0492	107.0493	107.0493	41	/
116	5-Methoxyindoleacetate	5.34	C_11_H_11_NO_3_	205.0733	[M − H]^−^	−0.85	204.0666	204.0664	117.0708, 130.0668, 160.0765, 204.0662	71	/
117	Lithospermic acid	5.35	C_27_H_22_O_12_	538.1106	[M + H – H_2_O]^+^	−2.30	521.1078	521.1066	203.0340, 249.0542, 267.0640, 277.0487, 281.0439, 295.0596, 323.0543, 341.0644, 493.1131, 521.1086	56.4	Salvia miltiorrhiza Bunge
118	Salvianolic acid B	5.37	C_36_H_30_O_16_	718.1528	[M − H]^−^	0.08	717.1461	717.1462	185.024, 277.0501, 279.0291, 293.0456, 295.0606, 321.0399, 339.0504, 519.0926, 717.1456	67.3	Salvia miltiorrhiza Bunge
119	Asiaticoside B	5.37	C_48_H_78_O_20_	974.5081	[M + Na]^+^	−1.82	957.5053	957.5036	433.3095, 439.3193, 451.3198, 469.3303, 471.1704, 487.3404, 597.3752, 633.3982, 649.3926, 795.4539	55.9	Centella asiatica (L.) Urban
120	Beta-D-Glucopyranoside, 2-((benzoyloxy)methyl)-4-hydroxyphenyl	5.39	C_20_H_22_O_9_	406.1258	[M + Na]^+^	−1.64	429.1156	429.1149	429.1149	52.7	/
121	Isololiolide	5.41	C_11_H_16_O_3_	196.1094	[M + H]^+^	−1.67	197.1172	197.1169	107.0857, 133.101, 135.1166, 161.0958, 179.1061, 197.1152	62.8	/
122	6″-O-Acetylglycitin	5.44	C_24_H_24_O_11_	488.1313	[M + H]^+^	−2.75	489.1391	489.1378	285.0751, 489.1368	55.2	Astragalus membranaceus
123	Asiaticoside B	5.49	C_48_H_78_O_20_	974.5081	[M − H]^−^	−0.03	973.5008	973.5008	101.0242, 161.0453, 247.0811, 469.1555, 503.3367, 973.5008	63.9	Centella asiatica (L.) Urban
124	Isoliquiritin	5.51	C_21_H_22_O_9_	418.1258	[M − H]^−^	−1.17	417.1191	417.1186	119.0498, 135.0086, 148.0163, 153.019, 175.0399, 254.0582, 255.0661, 417.1176, 417.1257	64	*Glycyrrhiza uralensi*s Fisch
125	Ononin	5.56	C_22_H_22_O_9_	430.1258	[M + H]^+^	−2.19	431.1337	431.1327	269.0803, 431.1286	62.5	Astragalus membranaceus
126	Oroxin A	5.57	C_21_H_20_O_10_	432.1051	[M − H]^−^	−1.54	431.0984	431.0977		56	
127	Licorice glycoside A	5.57	C_36_H_38_O_16_	726.2154	[M − H]^−^	0.38	725.2087	725.2090	119.0498, 134.037, 135.0085, 153.0188, 175.0398, 193.0499, 255.0663, 531.1516, 549.1627, 725.2092	67.8	*Glycyrrhiza uralensi*s Fisch
128	Isoononin	5.59	C_22_H_22_O_9_	430.1258	[M + FA – H]^−^	−0.90	475.1246	475.1242	252.0423, 267.066, 475.124	76.2	Astragalus membranaceus
129	Indole-3-acetic acid	5.61	C_10_H_9_NO_2_	175.0628	[M + H]^+^	−2.14	176.0706	176.0702	130.065, 149.0228, 176.0702	58.7	/
130	Salvianolic acid A	5.65	C_26_H_22_O_10_	494.1207	[M – H]^–^	−0.22	493.1140	493.1139	135.0452, 185.0242, 197.0451, 295.0608, 313.0713, 317.0298, 493.0486, 493.0592, 493.1143, 493.1212	56.2	Salvia miltiorrhiza Bunge
131	Scheffoleoside A	5.75	C_48_H_78_O_19_	958.5132	[M + H]^+^	−1.89	959.5210	959.5192	407.3304, 435.3241, 453.3352, 471.1745, 471.3463, 489.3565, 635.4145, 651.4111, 797.4689, 813.4622	61.3	Centella asiatica (L.) Urban
132	Madecassoside	5.78	C_48_H_78_O_19_	958.5132	[M − H]^−^	0.64	1003.5119	1003.5125	101.0243, 161.0455, 247.0822, 469.1559, 487.3423, 957.5062, 1003.5131	64.5	/
133	Licochalcone B	5.82	C_16_H_14_O_5_	286.0836	[M − H]^−^	−0.88	285.0768	285.0766	150.032, 270.0531, 285.0417, 285.0759	61.4	/
134	Lactiflorin	5.85	C_23_H_26_O_10_	462.1520	[M + NH_4_]^+^	−2.42	480.1865	480.1853	167.07, 301.1063, 463.1584	53.7	Astragalus membranaceus
135	3-O-a-Laminaribiosylplatycodigenin methyl ester	5.9	C_43_H_70_O_17_	858.4608	[M − H]^−^	0.66	857.4540	857.4546	193.0504, 323.0979, 487.3424, 811.45, 857.2621, 857.4526	65.8	/
136	7-O-Methylluteolin-6-C-beta-glucoside	5.94	C_16_H_14_O_7_	318.0734	[M – 2 OH – H]^−^	−0.94	299.0561	299.0558	256.0374, 269.0453, 284.0324, 284.0689, 299.0189, 299.0556	62	Astragalus membranaceus
137	Liquiritigenin	5.96	C_15_H_12_O_4_	256.0730	[M − H]^−^	0.49	255.0663	255.0664	119.05, 135.0086, 153.0191, 255.0661	61	*Glycyrrhiza uralensi*s Fisch
138	Isomucronulatol 7-O-glucoside	5.96	C_23_H_28_O_10_	464.1677	[M − H]^−^	−1.07	463.1616	463.1616	135.0447, 161.0238, 197.0451, 286.0828, 301.1077, 311.0555, 333.0247, 347.1852, 463.1616	61.6	Astragalus membranaceus
139	Isoliquiritigenin	5.98	C_15_H_12_O_4_	256.0730	[M + H]^+^	−2.34	257.0808	257.0802	137.023, 138.0264, 147.0433, 148.0471, 155.1064, 257.0802	65.3	*Glycyrrhiza uralensi*s Fisch
140	Luteolin	6	C_15_H_10_O_6_	286.0472	[M + H]^+^	−2.17	287.0550	287.0544	121.0285, 137.0231, 151.0388, 245.0803, 287.0535	45.6	*Glycyrrhiza uralensi*s Fisch、Centella asiatica (L.) Urban
141	Sebacic acid	6.02	C_10_H_18_O_4_	202.1200	[M − H]^−^	−0.68	201.1132	201.1131	89.0246, 116.9284, 139.1126, 183.1018, 201.1129	66.2	/
142	Lithospermic acid	6.05	C_27_H_22_O_12_	538.1106	[M – H] – 2OH	−0.60	519.0933	519.0930	109.0295, 185.0245, 277.0505, 279.03, 293.0457, 295.062, 321.0399, 339.0508, 519.0927	69.7	Salvia miltiorrhiza Bunge
143	Calycosin	6.17	C_16_H_12_O_5_	284.0679	[M − H]^−^	−0.77	283.0612	283.0610	268.0374, 283.0607	63.5	Astragalus membranaceus、*Glycyrrhiza uralensi*s Fisch
144	Syringaresinol	6.24	C_22_H_26_O_8_	418.1622	[M + H – H_2_O]+	−3.52	401.1594	401.1580	315.0855, 323.1257, 330.1090, 339.1208, 343.1151, 351.1218, 369.1295, 371.1465, 383.1478, 401.1588	60.7	/
145	Ixerisoside D	6.34	C_21_H_28_O_8_	408.1779	[M − H]^−^	3.71	407.1711	407.1727	407.1727	54.1	/
146	Isosalvianolic acid c	6.35	C_26_H_20_O_10_	492.1051	[M + H]^+^	−2.41	493.1129	493.1117	139.0387, 181.0488, 225.0536, 249.0536, 253.0477, 267.0646, 277.0485, 295.0592, 493.1134	68.5	Salvia miltiorrhiza Bunge
147	Phloretin	6.51	C_15_H_14_O_5_	274.0836	[M − H]^−^	−0.61	273.0768	273.0767	273.0767	40.9	/
148	Echinatin	6.72	C_16_H_14_O_4_	270.0887	[M + H]^+^	−2.93	271.0965	271.0957	107.0494, 121.0284, 123.044, 177.0543, 229.0856, 271.0592, 271.0954	57.1	Glycyrrhiza uralensis
149	Naringenin	6.72	C_15_H_12_O_5_	272.0679	[M − H]^−^	−1.44	271.0612	271.0608	109.029, 135.0087, 135.0448, 153.019, 165.0185, 211.1334, 271.0598	47	Astragalus membranaceus、*Glycyrrhiza uralensi*s Fisch
150	Bernardioside A	6.96	C_36_H_58_O_11_	666.3974	M + NH4	−1.85	684.4318	684.4305	325.1103, 359.0235, 405.3170, 429.0883, 433.3112, 451.3189, 469.3296, 487.3392, 586.5322, 669.2875	52.3	/
151	5-hydroxy-7,8-dimethoxy-6-methyl-3-(3′,4′-dihydroxybenzyl)chroman-4-one	6.97	C_19_H_20_O_7_	360.1204	[M – H_2_O – H]^−^	−2.51	341.1030	341.1022	89.0243, 119.0349, 179.0557, 253.1231, 297.1127, 341.1059	53.9	/
152	Pseudolaric Acid C	7.03	C_21_H_26_O_7_	390.1673	[M − H]^−^	1.95	389.1606	389.1613	389.1613	40.3	/
153	Isorhamnetin	7.07	C_16_H_12_O_7_	316.0578	[M − H]^−^	−0.73	315.0510	315.0508	109.0293, 300.0272, 315.0508	63.6	*Glycyrrhiza uralensi*s Fisch
154	4-Methoxymedicarpin	7.09	C_17_H_16_O_5_	300.0992	[M − H]^−^	−1.69	299.0925	299.0920	269.0815, 284.0324, 299.0552, 299.0916	54.3	Astragalus membranaceus
155	Asiaticoside D	7.09	C_48_H_78_O_18_	942.5183	[M + FA – H]^−^	1.49	987.5170	987.5184	101.0244, 125.0243, 143.0345, 161.0454, 247.0832, 367.1236, 469.1557, 471.3481, 941.5105, 987.5128	62.4	/
156	Tectorigenin	7.13	C_16_H_12_O_6_	300.0628	[M − H]^−^	−1.34	299.0561	299.0557	271.0252, 284.0323, 299.0183, 299.0549	56.6	Astragalus membranaceus
157	Astragaloside VI	7.22	C_47_H_78_O_19_	946.5132	[M + FA − H]^−^	−0.46	991.5120	991.5115	945.5055, 991.5023	62.3	/
158	Traumatic acid	7.3	C_12_H_20_O_4_	228.1356	[M − H]^−^	−0.92	227.1289	227.1287	165.1287, 181.0714, 183.1385, 184.1423, 227.1287	69.6	/
159	Licorice saponin G2	7.31	C_42_H_62_O_17_	838.3982	[M + H]^+^	−1.38	839.4060	839.4048	141.0179, 451.3198, 469.3304, 487.3407, 645.3594, 663.3655, 839.4042	73.7	Glycyrrhiza uralensis
160	pinocembrin	7.42	C_15_H_12_O_4_	256.0730	[M − H]^−^	−0.97	255.0663	255.0660	119.0501, 135.0087, 153.0192, 255.0662	65.6	*Glycyrrhiza uralensi*s Fisch
161	Isoliquiritigenin	7.44	C_15_H_12_O_4_	256.0730	[M + H]^+^	−2.27	257.0808	257.0803	137.0231, 147.0439, 257.0802	57	Astragalus membranaceus
162	Marmin	7.47	C_19_H_24_O_5_	332.1618	[M − H]^−^	−1.99	331.1551	331.1544	239.1438, 240.1466, 287.2012, 288.1676, 330.2366	42.8	/
163	Corosolic acid	7.67	C_30_H_48_O_4_	472.3547	[M + H – H_2_O]^+^	−2.85	455.3519	455.3506	109.1012, 123.117, 125.0964, 141.127, 143.1065, 297.2193, 419.3294, 437.3412, 455.225, 455.3501	51.6	Astragalus membranaceus
164	Astragaloside III	7.67	C_41_H_68_O_14_	784.4604	[M + FA – H]^−^	−0.08	829.4591	829.4590	829.4592	55.7	Astragalus membranaceus
165	Astragaloside IV	7.69	C_41_H_68_O_14_	784.4604	[M + H]^+^	−1.39	785.4682	785.4671	86.0966, 124.9996, 125.0961, 143.1065, 184.073, 437.3419, 455.3485	50	Astragalus membranaceus
166	Formononetin	7.7	C_16_H_12_O_4_	268.0730	[M − H]^−^	−0.00	267.0663	267.0663	252.0425, 267.0659	51.4	Astragalus membranaceus
167	Glycyrrhizic acid	7.77	C_42_H_62_O_16_	822.4032	[M + H]^+^	−0.97	823.4111	823.4103	453.3353, 471.346, 647.3774	68.6	*Glycyrrhiza uralensi*s Fisch
168	Alpha-Glycyrrhizin	7.78	C_42_H_62_O_16_	822.4032	[M − H]^−^	0.59	821.3965	821.3970	113.0243, 821.3904	66.4	/
169	Dihydroactinidiolide	8.06	C_11_H_16_O_2_	180.1145	[M + H]^+^	−1.40	181.1223	181.1221	107.0857, 111.0441, 135.1166, 163.1113, 181.1218	60.7	/
170	Soyasaponin Ba	8.12	C_48_H_78_O_19_	958.5132	[M + H]^+^	−2.71	959.5210	959.5184	405.3513, 423.3613, 441.3716, 501.1389, 581.3779, 599.3934, 617.4045, 635.4154, 797.4703, 959.5205	65.3	Centella asiatica (L.) Urban
171	Isomucronulatol	8.14	C_17_H_18_O_5_	302.1149	[M + H]^+^	−2.61	303.1227	303.1219	123.044, 133.0646, 149.059, 161.0595, 167.07, 181.0852, 193.0854, 303.0493	57.1	Astragalus membranaceus
172	Sandosaponin B	8.23	C_48_H_76_O_19_	956.4975	[M + H]^+^	−1.75	957.5054	957.5037	455.3502, 599.3959, 605.4351, 613.3696, 617.3990, 631.3812, 635.4091, 649.3883, 811.4476, 957.5079	66.2	/
173	Glicoricone	8.25	C_21_H_20_O_6_	368.1254	[M + H]^+^	−2.65	369.1333	369.1323	169.0494, 189.0906, 199.0749, 213.0883, 215.1062, 271.0597, 285.0745, 313.0697, 369.1301	71.5	Glycyrrhiza uralensis
174	Sinensetin	8.25	C_20_H_20_O_7_	372.1204	[M + H]^+^	−2.90	373.1282	373.1271	312.0972, 343.0803, 357.0961, 373.1268	60.1	*Glycyrrhiza uralensi*s Fisch
175	Licorice-saponin H2	8.27	C_42_H_62_O_16_	822.4032	[M + H]^+^	−1.13	823.4111	823.4101	453.3353, 471.3472, 647.3771	65.3	Glycyrrhiza uralensis
176	Soyasaponin Bb	8.33	C_48_H_78_O_18_	942.5183	[M + H]^+^	−1.49	943.5261	943.5247	441.3715, 581.3784, 599.3939, 605.4382, 617.4047, 635.4142, 781.4689, 797.4675, 943.5126, 943.5311	61.5	Astragalus membranaceus
177	Soyasaponin III	8.33	C_42_H_68_O_14_	796.4604	[M + H]^+^	−1.09	797.4682	797.4673	247.2049, 383.3297, 405.3498, 423.3611, 441.3716, 581.3814, 599.3936, 617.4048, 635.4114, 797.4686	60.7	Astragalus membranaceus
178	TMC-58B	8.56	C_25_H_26_N_2_O_3_	402.1938	[M—H]-	−1.70	401.1871	401.1864	120.0453, 146.0609, 267.1139, 280.1339, 401.1867	61.4	/
179	Medicarpin	8.56	C_16_H_14_O_4_	270.0887	[M + H]^+^	−2.00	271.0965	271.0959	123.0441, 137.0596, 161.0592, 271.0956	51.4	Astragalus membranaceus、 *Glycyrrhiza uralensi*s Fisch
180	Pulsatilla saponin D	8.64	C_47_H_76_O_17_	912.5077	[M + H]^+^	−1.48	913.5155	913.5142	323.0973, 405.3507, 423.3611, 441.3716, 599.3931, 605.4415, 617.4045, 635.4149, 767.4562, 781.4749	44.8	Astragalus membranaceus
181	Acacetin	8.77	C_16_H_12_O_5_	284.0679	[M − H]^−^	−1.14	283.0612	283.0609	268.0377, 283.0266, 283.0607	59.7	Glycyrrhiza uralensis、Astragalus membranaceus、Abelmoschus manihot
182	Rishitin	8.83	C_14_H_22_O_2_	222.1614	[M + FA – H]^−^	−1.55	267.1603	267.1598	223.1702, 267.0663, 267.1598	59	
183	AstragalosideII	8.91	C_43_H_70_O_15_	826.4709	[M + FA – H]^−^	0.22	871.4697	871.4699	112.9856, 871.4694	59.5	Astragalus membranaceus
184	Paris saponin VII	8.97	C_51_H_82_O_21_	1030.5343	[M + H]^+^	−3.61	1031.5421	1031.5384	1031.5384	43.5	/
185	14alpha-hydroxy Sprengerinin C	9	C_44_H_70_O_17_	870.4608	[M − H]^−^	−0.1	869.4540	869.4539	869.4539	40.4	/
186	Dehydrosoyasaponin I	9.07	C_48_H_76_O_18_	940.5026	[M + H]^+^	−1.24	941.5104	941.5093	309.1171, 421.3458, 439.3557, 457.3661, 597.3780, 603.4197, 615.3898, 633.4066, 795.4581, 941.5150	66	/
187	Glycycoumarin	9.08	C_21_H_20_O_6_	368.1254	[M − H]^−^	−1.84	367.1187	367.1180	139.0398, 297.0396, 309.0399, 367.1191	59.1	*Glycyrrhiza uralensi*s Fisch
188	Glyasperin C	9.22	C_21_H_24_O_5_	356.1618	[M + H]^+^	−3.01	357.1697	357.1686	165.0541, 179.0690, 221.1171, 235.1313, 289.1072, 294.1197, 301.1062, 312.1293, 357.1621, 357.1681	63.8	Glycyrrhiza uralensis
189	Artepillin A	9.27	C_19_H_24_O_4_	316.1669	[M − H]^−^	−1.94	315.1602	315.1596	225.1642, 271.1699, 315.1595	57	/
190	Arjunolic acid	9.39	C_30_H_48_O_5_	488.3496	[M + FA – H]^−^	−0.67	533.3484	533.3480	487.3423, 533.3499	66.5	Centella asiatica (L.) Urban
191	Angenomalin	9.53	C_14_H_12_O_3_	228.0781	[M + H]^+^	−1.56	229.0859	229.0856	175.039, 187.0388, 229.0854	62.4	
192	Trijuganone B	9.54	C_18_H_16_O_3_	280.1094	[M + FA – H]^−^	−1.89	325.1082	325.1076	281.1179, 325.1076	68	Salvia miltiorrhiza Bunge
193	Astragaloside I	9.57	C_45_H_72_O_16_	868.4815	M + Na	−1.26	891.4713	891.4702	711.4077, 891.4701	50.6	Astragalus membranaceus
194	Hederagenin	9.59	C_30_H_48_O_4_	472.3547	[M + H – H_2_O]^+^	-2.19	455.3519	455.3509	109.1015, 123.1169, 125.0959, 141.1271, 143.1064, 297.2205, 419.3307, 437.341, 455.351	63.2	Astragalus membranaceus
195	Danshenxinkun A	9.6	C_18_H_16_O_4_	296.1043	[M − H]^−^	−1.65	295.0976	295.0971	237.0918, 265.087, 267.1019, 295.0968	63.3	Salvia miltiorrhiza Bunge
196	Licoisoflavone A	9.62	C_20_H_18_O_6_	354.1098	[M − H]^−^	−1.56	353.1031	353.1025	125.0242, 353.1025	62.5	*Glycyrrhiza uralensi*s Fisch
197	Neoglycyrol	9.83	C_21_H_18_O_6_	366.1098	[M − H]^−^	−2.83	365.1031	365.1020	295.0237, 297.1132, 307.0244, 365.102	56.5	Glycyrrhiza uralensis
198	3-Hydroxydodecanoic acid	9.87	C_12_H_24_O_3_	216.1720	[M − H]^−^	−1.26	215.1653	215.1650	59.0137, 116.9287, 160.9347, 215.1292, 215.1647	46.3	/
199	Beta-Kudinlactone	9.89	C_30_H_46_O_5_	486.3340	[M − H]^−^	−0.96	485.3272	485.3268	485.3277	50.7	Abelmoschus manihot
200	Dihydrotanshinone I	10.19	C_18_H_14_O_3_	278.0937	[M + H]^+^	−1.76	279.1016	279.1011	149.0231, 233.0957, 261.0905, 279.1009	62.5	Salvia miltiorrhiza Bunge
201	21-Hydroxygypsogenin	10.27	C_30_H_46_O_5_	486.3340	[M − H]^−^	−1.11	485.3272	485.3267	485.3275	53.7	Glycyrrhiza uralensis
202	9,10-Dihydroxystearic acid	10.39	C_18_H_36_O_4_	316.2608	[M − H]^−^	−1.89	315.2541	315.2535	315.2535	52.6	/
203	Neocryptotanshinone	10.44	C_19_H_22_O_4_	314.1513	[M + H]^+^	−2.50	315.1591	315.1583	251.1427, 279.1374, 297.1476, 315.1581	59.7	Salvia miltiorrhiza Bunge
204	( ±)9-HODE	10.81	C_18_H_32_O_3_	296.2346	[M − H]^−^	−1.80	295.2279	295.2273	277.2161, 295.2272	70.5	Astragalus membranaceus
205	Tanshinone I	10.89	C_18_H_12_O_3_	276.0781	[M + H]^+^	−1.97	277.0859	277.0854	249.0905, 277.0853	62.6	Salvia miltiorrhiza Bunge
206	Diosgenin glucoside	11.22	C_33_H_52_O_8_	576.3656	[M − H]^−^	0.45	577.3735	577.3738	577.3738	52	
207	Licorice-saponin H2_qt	11.46	C_30_H_46_O_4_	470.3391	[M + H]^+^	−1.89	471.3469	471.3460	471.3466	59.1	/
208	18alpha-Glycyrrhetinic acid	11.49	C_30_H_46_O_4_	470.3391	[M − H]^−^	−1.58	469.3323	469.3316	469.0264, 469.3314	55.6	*Glycyrrhiza uralensi*s Fisch
209	Tanshinone IIA	11.67	C_19_H_18_O_3_	294.1250	[M + H]^+^	−1.97	295.1329	295.1323	249.1268, 277.1215, 295.1322	61.9	Salvia miltiorrhiza Bunge
210	3-Hydroxypalmitic acid	12.22	C_16_H_32_O_3_	272.2346	[M − H]^−^	−1.06	271.2279	271.2276	225.2221, 271.2273	66.7	/
211	Docosa-4,7,10,13,16,19-hexaenoic acid	12.52	C_22_H_32_O_2_	328.2397	[M − H]^−^	−1.4	327.2330	327.2325	229.1957, 283.2427, 327.2324	50.6	/

**Table 4:** Analysis and identification of prototype compounds in mouse plasma of HY-I based on UPLC-Q-TOF–MS

No.	No. (HY-I)	English name	RT	Formula	Calc. MW	Adducts	Error (ppm)	Theoretical mass (m/z)	Experimental mass (m/z)	MS^2^ (m/z)
P1	24	Pinitol	1.05	C_7_H_14_O_6_	194.0785	[M + FA-H]^−^	−1.19	239.0773	239.0770	193.0703, 195.0288, 195.1377, 201.9223, 238.8911, 239.0168, 239.0230, 239.0551, 239.0626, 239.0770
P2	45	Ser-Leu	2.6	C_9_H_18_N_2_O_4_	218.1261	[M + H]^+^	−1.47	219.1339	219.1336	60.0449, 86.0968, 132.1017, 173.128, 201.122, 203.1425, 205.1583, 219.1116, 219.1333
P3	79	Doryphornine	4.62	C_11_H_11_NO_3_	205.0733	[M + FA-H]^−^	−0.98	250.0721	250.0719	88.0403, 115.0036, 132.0301, 135.0449, 250.0717
P4	83	Aloe-emodin-8-O-beta-D-glucopyranoside	4.79	C_21_H_20_O_10_	432.1051	[M − H]^−^	−1.41	431.0984	431.0978	113.0244, 139.1761, 153.0183, 175.0242, 208.4511, 208.4739, 209.3472, 255.0657, 431.0968
P5	85	Taxifolin 7-O-rhamnoside	4.8	C_21_H_22_O_11_	450.1157	[M + H-H_2_O] +	−2.12	433.1129	433.1120	313.0695, 323.0909, 337.0698, 349.0700, 361.0684, 367.0800, 379.0804, 397.0909, 415.1018, 433.1120
P6	89	( +)-3,4′,5,7-Flaventetrol	4.86	C_15_H_14_O_5_	274.0836	[M + H-H_2_O] +	−2.00	257.0808	257.0803	137.0231, 147.0438, 257.0804
P7	93	3,4-Dimethoxybenzyl alcohol	4.96	C_9_H_12_O_3_	168.0781	[M + NH_4_]^+^	−1.43	186.1125	186.1122	83.086, 111.0808
P8	97	Nepitrin	5.01	C_22_H_22_O_12_	478.1106	[M − H − H_2_O]^−^	−0.47	459.0933	459.0931	459.0931
P9	101	Kaempferol 3-O-D-galactoside	5.03	C_21_H_20_O_11_	448.1000	[M − H]^−^	−1.49	447.0933	447.0926	255.0295, 284.0323, 447.0927
P10	103	Trifolin	5.04	C_21_H_20_O_11_	448.1000	[M + H]^+^	−2.01	449.1078	449.1069	287.0544, 288.0575, 303.049
P11	106	Astilbin	5.12	C_21_H_22_O_11_	450.1157	[M + H-H_2_O] +	−2.27	433.1129	433.1119	271.0594, 272.063, 433.1078
P12	116	5-Methoxyindoleacetate	5.34	C_11_H_11_NO_3_	205.0733	[M − H]^−^	−0.85	204.0666	204.0664	117.0708, 130.0668, 160.0765, 204.0662
P13	123	Asiaticoside B	5.49	C_48_H_78_O_20_	974.5081	[M − H]^−^	−0.03	973.5008	973.5008	101.0242, 161.0453, 247.0811, 469.1555, 503.3367, 973.5008
P14	126	Oroxin A	5.57	C_21_H_20_O_10_	432.1051	[M − H]^−^	−1.54	431.0984	431.0977	431.0977
P15	151	5-hydroxy-7,8-dimethoxy-6-methyl-3-(3′,4′-dihydroxybenzyl)chroman-4-one	6.97	C_19_H_20_O_7_	360.1204	[M—H_2_O—H]^−^	−2.51	341.1030	341.1022	89.0243, 119.0349, 179.0557, 253.1231, 297.1127, 341.1059
P16	152	Pseudolaric acid C	7.03	C_21_H_26_O_7_	390.1673	[M − H]^−^	1.95	389.1606	389.1613	389.1613
P17	162	Marmin	7.47	C_19_H_24_O_5_	332.1618	[M − H]^−^	−1.99	331.1551	331.1544	239.1438, 240.1466, 287.2012, 288.1676, 330.2366
P18	168	Alpha-Glycyrrhizin	7.78	C_42_H_62_O_16_	822.4032	[M − H]^−^	0.59	821.3965	821.3970	113.0243, 821.3904
P19	182	Rishitin	8.83	C_14_H_22_O_2_	222.1614	[M + FA—H]^−^	−1.55	267.1603	267.1598	223.1702, 267.0663, 267.1598
P20	189	Artepillin A	9.27	C_19_H_24_O_4_	316.1669	[M − H]^−^	−1.94	315.1602	315.1596	225.1642, 271.1699, 315.1595
P21	190	Arjunolic acid	9.39	C_30_H_48_O_5_	488.3496	[M + FA—H]^−^	−0.67	533.3484	533.3480	487.3423, 533.3499
P22	198	3-Hydroxydodecanoic acid	9.87	C_12_H_24_O_3_	216.1720	[M − H]^−^	−1.26	215.1653	215.1650	59.0137, 116.9287, 160.9347, 215.1292, 215.1647
P23	199	Beta-Kudinlactone	9.89	C_30_H_46_O_5_	486.3340	[M − H]^−^	−0.96	485.3272	485.3268	485.3277
P24	201	21-Hydroxygypsogenin	10.27	C_30_H_46_O_5_	486.3340	[M − H]^−^	−1.11	485.3272	485.3267	485.3275
P25	202	9,10-Dihydroxystearic acid	10.39	C_18_H_36_O_4_	316.2608	[M − H]^−^	−1.89	315.2541	315.2535	315.2535
P26	204	Neocryptotanshinone	10.44	C_19_H_22_O_4_	314.1513	[M + H]^+^	−2.50	315.1591	315.1583	251.1427, 279.1374, 297.1476, 315.1581
P27	206	Diosgenin glucoside	11.22	C_33_H_52_O_8_	576.3656	[M − H]^−^	0.45	577.3735	577.3738	577.3738
P28	207	Licorice-saponin H2_qt	11.46	C_30_H_46_O_4_	470.3391	[M + H]^+^	−1.89	471.3469	471.3460	471.3466
P29	208	18alpha-Glycyrrhetinic acid	11.49	C_30_H_46_O_4_	470.3391	[M − H]^−^	−1.58	469.3323	469.3316	469.0264, 469.3314

**Table 5:** Analysis and identification of metabolites in mouse plasma of HY-I based on UPLC-Q-TOF–MS

No.	Metabolites	RT(min)	Formula	Calc. MW	Adducts	Mass Error (ppm)	m/z	Score	Parent compound	Transformations
M1	10-Hydroxymajoroside_M1	2.28	C_10_H_12_O_9_S	308.0196	[M + FA-H]^−^	3.99	353.0196	48.8	10-Hydroxymajoroside	Deglycosidation, hydrolysis, sulfation
M2	5,7-Dihydroxy-4-oxo-4H-chromene-2-carboxylic acid_M2	3.81	C_17_H_16_O_13_	428.0585	[2 M-H]^−^	0.47	855.1113	43	5,7-Dihydroxy-4-oxo-4H-chromene-2-carboxylic acid	Hydroxylation, glucuronidation, methylation
M3	5,7-Dihydroxy-4-oxo-4H-chromene-2-carboxylic acid_M1	3.83	C_10_H_6_O_7_	238.0108	[M + FA-H]^−^	1.43	283.0099	41.9	5,7-Dihydroxy-4-oxo-4H-chromene-2-carboxylic acid	Hydroxylation
M4	3-Coumaric acid_M1	4.28	C_9_H_8_O_6_S	244.0036	[M − H]^−^	−1.10	242.9966	41.1	3-Coumaric acid	Sulfation
M5	3,4-Dihydroxybenzaldehyde_M1	4.41	C_7_H_6_O_2_	122.0362	[M-H_2_O-H]^−^	2.54	105.0338	57.1	3,4-Dihydroxybenzaldehyde	Dehydroxylation
M6	Beta-D-Glucopyranoside, 2-((benzoyloxy)methyl)-4-hydroxyphenyl_M1	4.42	C_9_H_9_NO_3_	179.0577	[M − H]^−^	−0.99	178.0508	54.4	Beta-D-Glucopyranoside, 2-((benzoyloxy)methyl)-4-hydroxyphenyl	Hydrolysis, glycination
M7	m-Methoxyphenol_M1	4.73	C_7_H_8_O_5_S	204.0087	[M − H]^−^	−0.53	203.0019	53.9	m-Methoxyphenol	Sulfation
M8	Luteolin_M1	4.75	C_21_H_18_O_15_S	542.0361	[M − H]^−^	−0.71	541.0290	54.3	Luteolin	Glucuronidation, sulfation
M9	Methyl protocatechuate_M1	4.93	C_9_H_10_O_7_S	262.0142	[M − H]^−^	−1.41	261.0071	54.5	Methyl protocatechuate	Methylation, sulfation
M10	Indoleacetic acid_M1	4.99	C_11_H_11_NO_4_	221.0682	[M-H_2_O-H]^−^	−0.60	202.0508	40.8	Indoleacetic acid	Hydroxylation, hydroxylation, methylation
M11	5-hydroxy-7,8-dimethoxy-6-methyl-3-(3′,4′-dihydroxybenzyl)chroman-4-one_M1	6.17	C_19_H_22_O_8_	378.1309	[M + FA-H]^−^	−0.96	423.1293	49.4	5-hydroxy-7,8-dimethoxy-6-methyl-3-(3′,4′-dihydroxybenzyl)chroman-4-one	Reduction, hydroxylation
M12	Phloretin_M1	6.25	C_15_H_14_O_8_S	354.0404	[M − H]^−^	−1.40	353.0332	59.6	Phloretin	Sulfation
M13	Ixerisoside D_M1	7.45	C_15_H_16_O_3_	244.1094	[M − H]^−^	−1.36	243.1023	44.2	Ixerisoside D	Deglycosidation, oxidation
M14	14alpha-hydroxy Sprengerinin C_M1	9.39	C_27_H_42_O_5_	446.3027	[M − H]^−^	−0.71	445.2956	55	14alpha-hydroxy Sprengerinin C	Deglycosidation, hydroxylation
M15	Paris saponin VII_M1	9.74	C_27_H_42_O_5_	446.3027	[M + H]^+^	−1.88	447.3097	56	Paris saponin VII	Deglycosidation, hydroxylation
M16	3-Hydroxypalmitic acid_M2	9.79	C_16_H_30_O_4_	286.2139	[M-H_2_O-H]^−^	−0.76	267.1964	46.8	3-Hydroxypalmitic acid	Oxidation, hydroxylation
M17	Docosa-4,7,10,13,16,19-hexaenoic acid_M1	10.73	C_22_H_32_O_3_	344.2346	[M − H]^−^	−1.55	343.2273	45.5	Docosa-4,7,10,13,16,19-hexaenoic acid	Hydroxylation
M18	3-Hydroxypalmitic acid_M1	10.77	C_16_H_30_O_4_	286.2138	[M-H_2_O-H]^−^	−1.27	267.1962	42.5	3-Hydroxypalmitic acid	Oxidation, hydroxylation
M19	Docosa-4,7,10,13,16,19-hexaenoic acid_M2	11	C_22_H_32_O_3_	344.2346	[M − H]^−^	−1.53	343.2273	53.8	Docosa-4,7,10,13,16,19-hexaenoic acid	Hydroxylation

**Table 6:** The main antibodies used in the present study

Reagent	Manufacturer	Item no
Protein ladder (1—180 kDa)	Thermo Fisher Scientific	26616
Anti-Ki-67 (D3B5) Rabbit mAb	Cell Signaling Technology	9129S
Anti-p21 Rabbit pAb	Wanlei bio	WL0362
Anti-β-actin antibody	Abcam	ab179467
Anti-p16 Rabbit pAb	Abcam	ab51243
Anti-TGFβ1 Rabbit pAb	Abclona	A15103
Anti-IL-1B Rabbit pAb	Wanlei bio	WL02257
Anti-STAT3 Rabbit pAb	Wanlei bio	WL01836
Anti-p-STAT3 Rabbit pAb	Wanlei bio	WLP0624
Anti-NF-κB Rabbit pAb	Wanlei bio	WL01980
Anti- p-NF-κB Rabbit pAb	Wanlei bio	WL02169
Anti-NLRP3 Rabbit pAb	Abmart	p60622r3f
Anti-Caspase-1/Cleaved Caspase-1 Rabbit pAb	Wanlei bio	WL03450
Anti-IL18 Rabbit pAb	Wanlei bio	WL01127
Anti-FN/Fibronectin Rabbit pAb	Wanlei bio	WL00712a
Anti-Collagen1 Rabbit pAb	Abclonal-	A1352
Anti-Vimentin Rabbit pAb	Abcam	ab8978
α-smooth muscle actin rabbit mAb	Abclonal	A17910
Anti-rabbit IgG, HRP-linked Antibody	Cell signaling technology	7074S
Anti-mouse IgG, HRP-linked Antibody	Cell signaling technology	7076S

**Table 7:** Molecular Docking

Target Gene	PBD ID	Ligand	PubChem ID	Binding Energy(kcal·mol-1)
STAS3	1BG1	Phloretin	4788	−3.66
		Luteolin	5280445	−5.19
		Pseudolaric acid C	6440704	−5.29
		18alpha-glycyrrhetinic acid	73398	−7.15
		Arjunolic acid	73641	−5.25
NLRP3	6NPY	Phloretin	4788	−5.18
		Luteolin	5280445	−5.99
		Pseudolaric acid C	6440704	−6.78
		18alpha-glycyrrhetinic acid	73398	−8.82
		Arjunolic acid	73641	−7.37
NFKB	1MY7	Phloretin	4788	−4.07
		Luteolin	5280445	−4.83
		Pseudolaric acid C	6440704	−5.59
		18alpha-glycyrrhetinic acid	73398	−7.16
		Arjunolic acid	73641	−5.46

### HY-I ameliorates renal senescence and pathological injury in aged mice

**Fig. 2:**
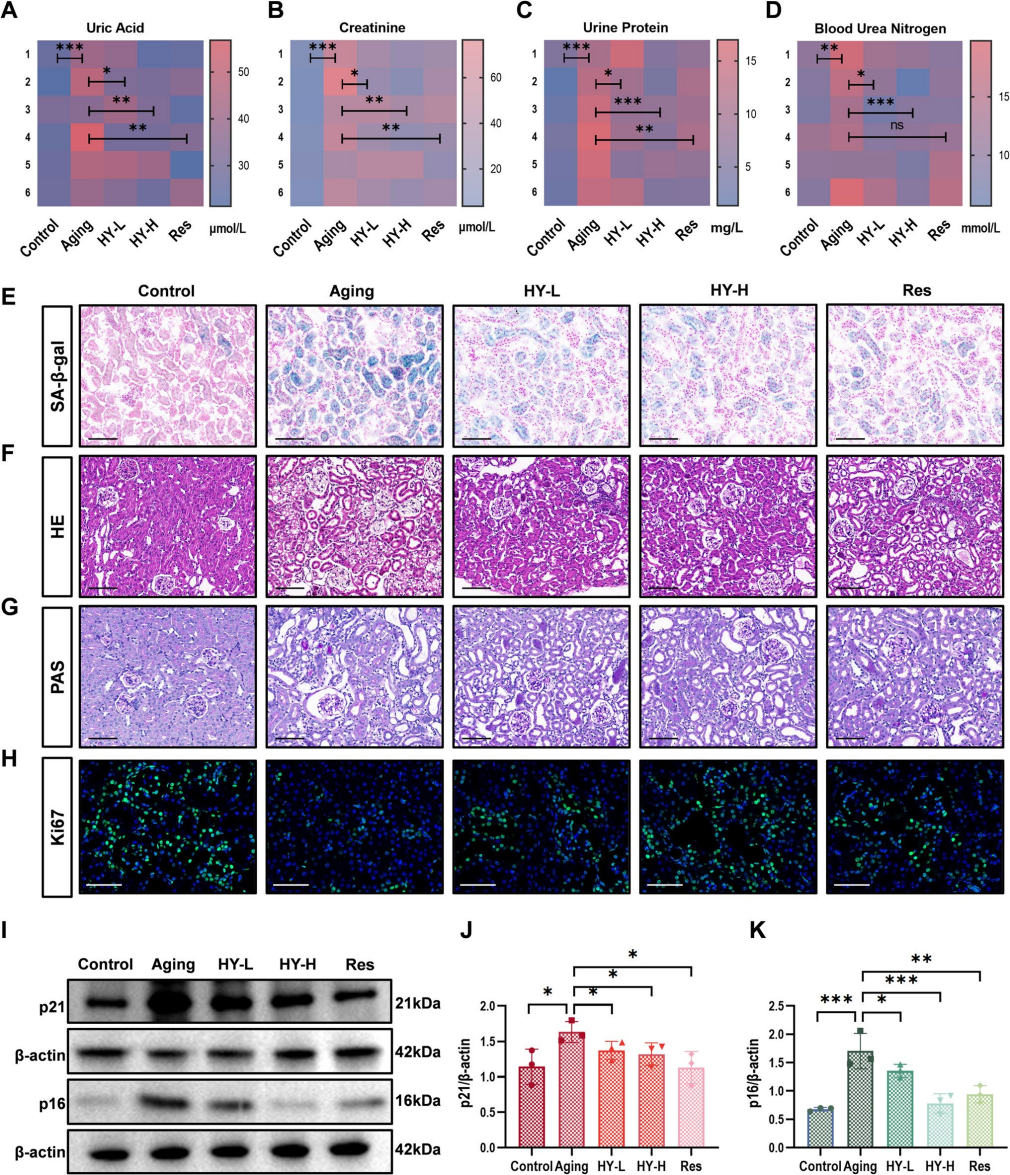
HY-I Ameliorates renal senescence and pathological injury in aged mice. **A**–**D** Analysis of renal function parameters (creatinine, BUN, urinary protein, uric acid) across experimental groups. n = 6. One-way ANOVA was used. * indicates comparison with the aging model group; *p < 0.05, **p < 0.01, ***p < 0.001; ns indicates no significant difference. **E** SA-β-gal staining of renal tissues. Scale bars, 100 μm. **F**, **G** Renal histopathology assessed by H&E and PAS staining. Scale bars, 100 μm. **H** Immunofluorescent detection of proliferation marker Ki67. Scale bars, 50 μm. **I**–**K** Representative immunoblots of p21 and p16 expression. n = 3. One-way ANOVA was used. * indicates comparison with the aging model group; *p < 0.05, **p < 0.01, ***p < 0.001; ns indicates no significant difference

HY-I efficacy was evaluated through renal function parameters, senescence biomarkers, and histopathological analysis. Intervention with HY-I significantly reduced serum urea nitrogen, creatinine, uric acid, and urinary protein levels in aged mice (Fig. 2A–D), indicating improved age-related renal dysfunction.

SA-β-gal staining demonstrated markedly decreased senescent cells in renal tubules following HY-I treatment (Fig. 2E). Histological assessment revealed that HY-I alleviated age-induced tubular pathologies including vacuolar degeneration, tubular atrophy, and brush border loss, as evidenced by H&E and PAS staining (Fig. 2F, G).

Furthermore, HY-I treatment restored the proliferation marker Ki67 (Fig. 2H) while suppressing senescence markers p21 and p16 (Fig. 2I–K). Collectively, these findings demonstrate HY-I effectively mitigates renal senescence in aged mice.

### Network pharmacology analysis of HY-I on renal senescence

**Fig. 3:**
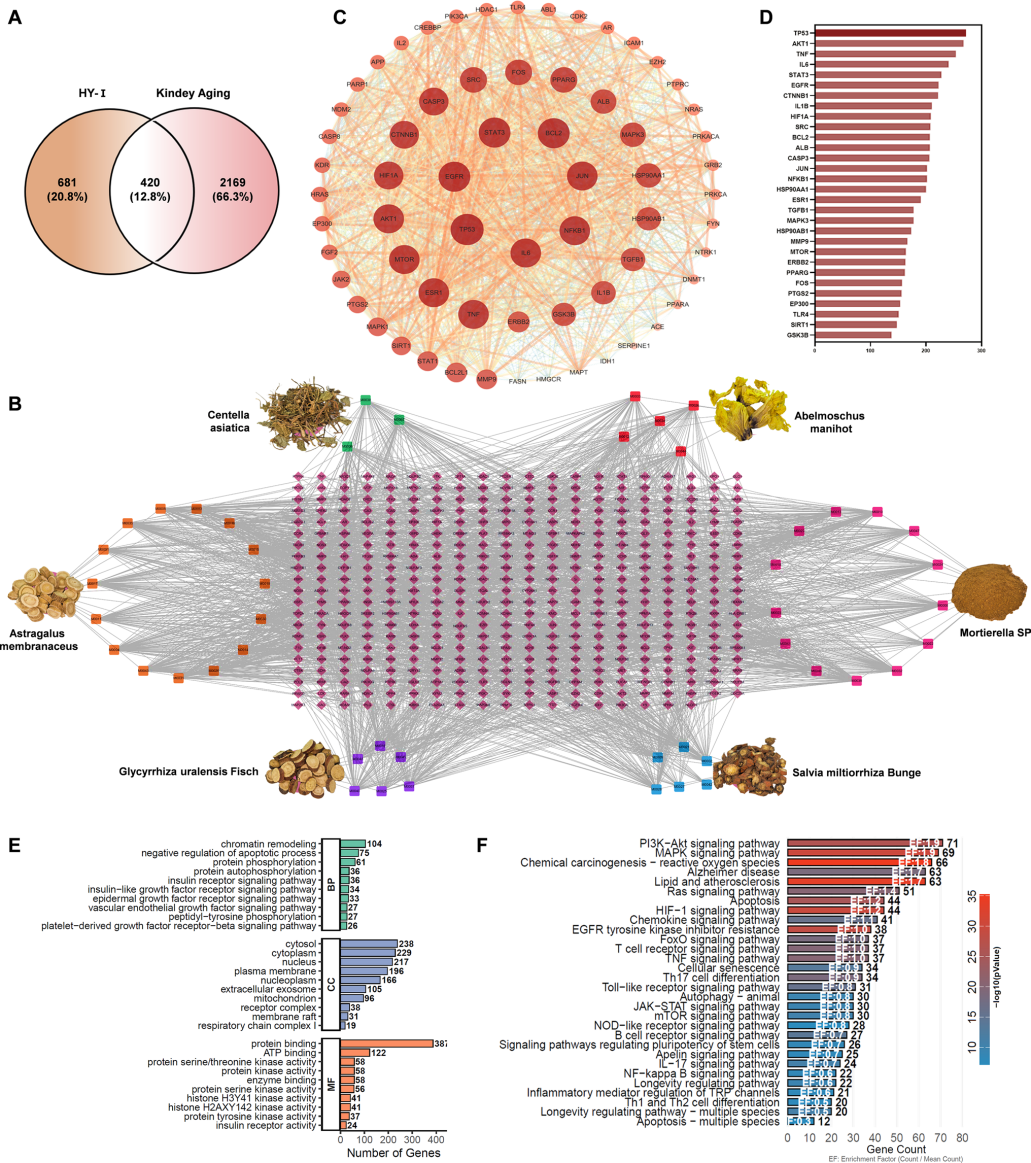
Network pharmacology analysis of HY-I on renal senescence. **A** Venn diagram of HY-I targets versus kidney aging (KA)-related genes. **B** HY-I-KA pharmacological network of compound-target interactions. **C** Core target PPI network after screening. **D** Top 30 genes in PPI network ranked by degree centrality. **E** GO enrichment analysis. **F** KEGG pathway enrichment

48 bioactive components of HY-I detectable in serum were selected. After integration and deduplication of the corresponding targets, a total of 681 unique proteins were identified. Venn analysis revealed 420 overlapping targets shared by HY-I and renal aging (Fig. 3A). Following serum-adsorbed compound attribution, a “herb–compound–target–disease” network was constructed (Fig. 3B).

The 420 common targets were further analyzed using the STRING database for protein–protein interactions (PPI). Topological analysis in Cytoscape 3.7.1 was performed based on parameters of betweenness centrality, closeness centrality, and degree. Using the median degree as a threshold, 65 core targets were selected for PPI network visualization, where node color and size were scaled according to degree values (Fig. 3C). The top 30 targets, ranked by degree, included IL-6, STAT3, and IL-1β, which are known to play essential roles in inflammation-associated senescence.

Enrichment analysis of the top 30 GO terms ranked by *P*-value revealed significant enrichment in biological processes such as chromatin remodeling and protein phosphorylation. The main cellular components were the cytoplasm and receptor complexes, while the predominant molecular functions involved protein and enzyme binding (Fig. 3E). Integrating the top 30 key targets identified as functionally important, KEGG pathway analysis further indicated that the JAK-STAT, NOD-like receptor, and NF-κB signaling pathways were prominently involved in the aging process (Fig. 3F).

### HY-I attenuates SASP expression in aged renal tubules

Renal tubular epithelial cells, being the most abundant and metabolically active renal cells, are highly susceptible to senescence. SASP—comprising proinflammatory cytokines, chemokines, and growth factors—drives localized inflammation and fibrosis. Network pharmacology predicted HY-I targets SASP components (IL-6, TGFβ1, IL-1β, MMP9) and inflammatory regulators (NF-κB, STAT3, NLRP3), suggesting its anti-senescence mechanism.

To validate HY-I’s effects on tubular SASP, drug-containing serum was applied to senescent tubular cells. SASP expression was assessed via IHC, WB, and ELISA in murine renal tissues and cells.

**Fig. 4:**
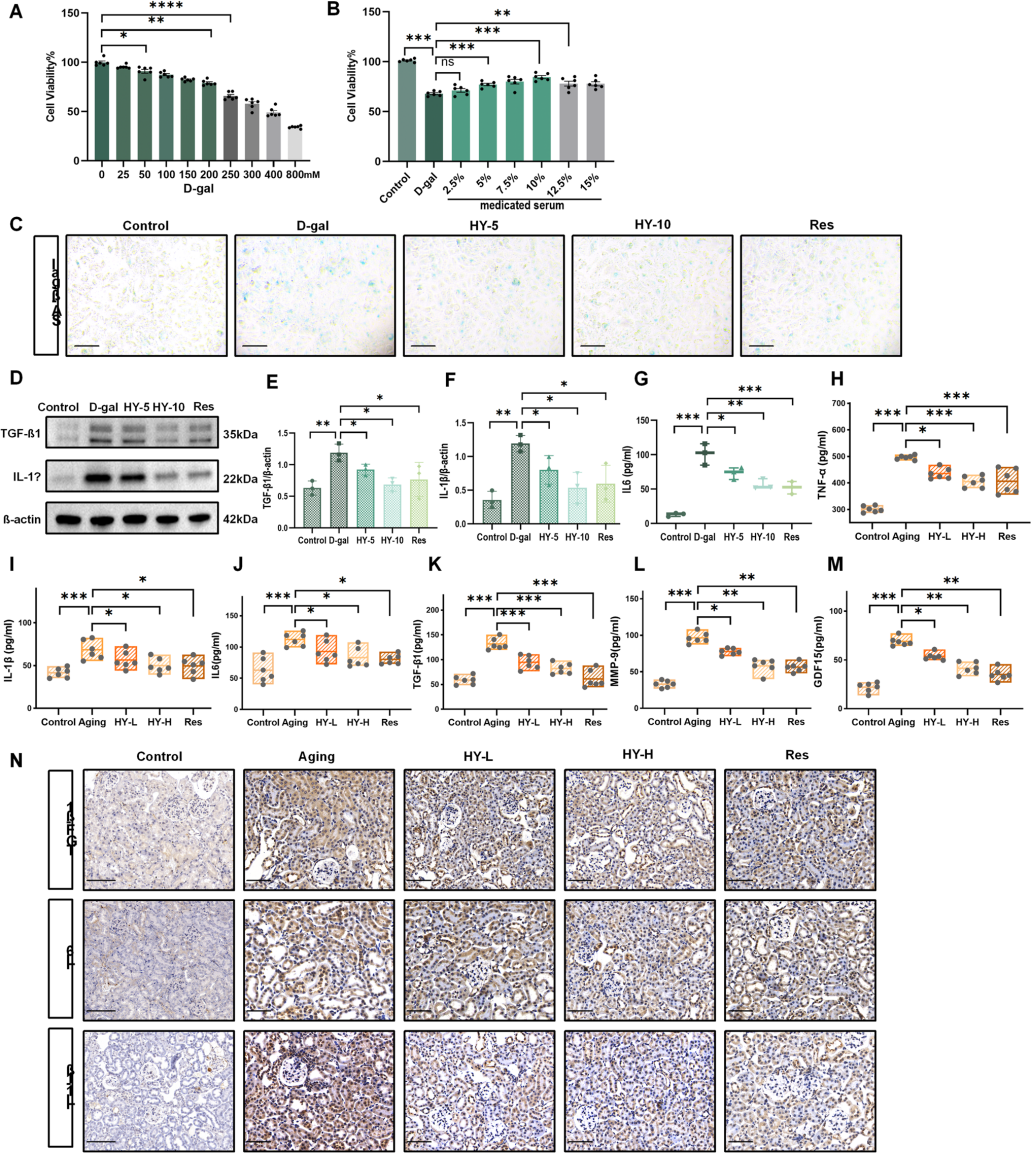
HY-I attenuates SASP expression in aged renal tubules. **A**, **B** CCK-8 assay: Cytotoxicity of D-gal in HK-2 cells and rescue by HY-I-containing serum. (n = 6). **C** SA-β-Gal staining of senescent cells. Scale bars, 100 μm. **D**–**F** WB analysis of TGF-β1 and IL-1β expression in D-gal-treated HK-2 cells with HY-I-containing serum intervention. (n = 3). **G** IL-6 levels in cell supernatants by ELISA. (n = 3). **H**–**M** Renal tissue SASP factors (TNF-α, IL-1β, IL-6, TGF-β1) quantified by ELISA. (n = 6). One-way ANOVA was used. * indicates comparison with the aging model group; *p < 0.05, **p < 0.01, ***p < 0.001; ns indicates no significant difference. **N** Representative immunohistochemical images of renal SASP factors (IL-1β, IL-6, TGF-β1). Scale bars, 100 μm

CCK-8 assays identified 200 mM D-galactose as optimal for HK-2 senescence induction (Fig. 4A). Drug-containing serum (5%-10%) restored cellular viability (Fig. 4B). Subsequent experiments used 5% and 10% concentrations. HY-I-containing serum reduced SA-β-gal-positive cells (Fig. 4C). WB/ELISA analyses of cell lysates, supernatants, and renal homogenates demonstrated HY-I-mediated suppression of SASP factors (Fig. 4D–M). IHC confirmed decreased TGF-β1, IL-6, and IL-1β expression in HY-I-treated kidneys (Fig. 4N). These results indicate HY-I concurrently mitigates tubular senescence and renal SASP.

### HY-I attenuates tubular inflammation and senescence via STAT3/NF-κB/NLRP3 axis

**Fig. 5:**
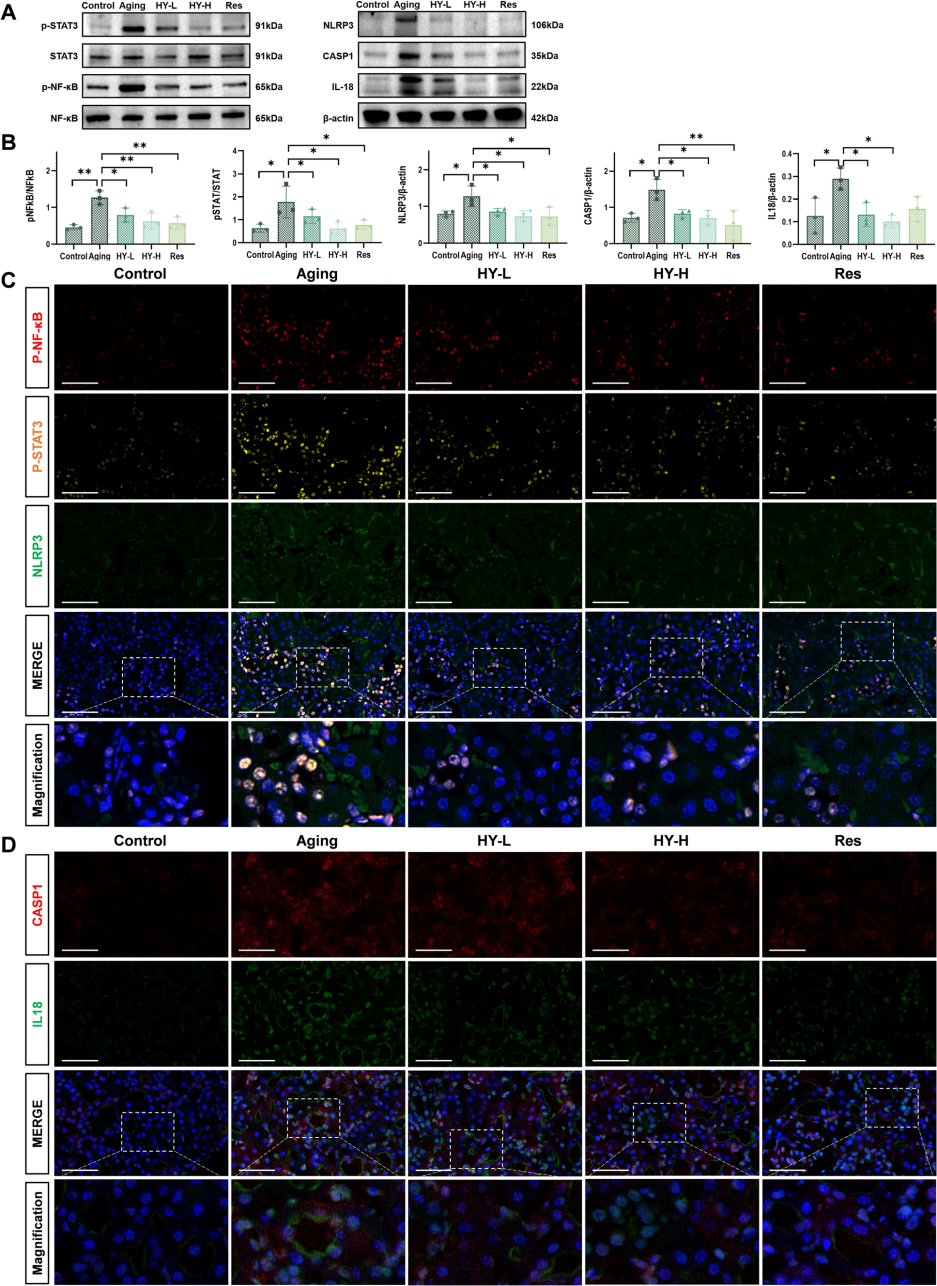
HY-I attenuates tubular inflammation and senescence via STAT3/NF-κB/NLRP3 Axis. **A**, **B** Western blot analysis of STAT3, p-STAT3, NF-κB, p-NF-κB, NLRP3, CASP1, and IL-18 expression in renal tissues. n = 3. One-way ANOVA was used. * indicates comparison with the aging model group; *p < 0.05, **p < 0.01, ***p < 0.001; ns indicates no significant difference. **C** Multiplex immunofluorescence of renal sections: p-NF-κB (red), p-STAT3 (yellow), NLRP3 (green), DAPI (blue). Scale bars, 50 μm. **D** Immunofluorescence of CASP1 (red) and IL-18 (green). Scale bars, 50 μm

Integrated network pharmacology and experimental validation suggested HY-I modulates inflammation through STAT3-NF-κB-NLRP3 signaling and downstream effectors (CASP1, IL-18). Western blotting demonstrated HY-I significantly reduced phosphorylation of NF-κB and STAT3, while downregulating NLRP3, CASP1, and IL-18 protein expression in renal tissues (Fig. 5A, B).

Multiplex immunofluorescence revealed enhanced nuclear translocation of p-NF-κB and p-STAT3 with concomitant upregulation of NLRP3 and co-expression of CASP1/IL-18 during renal aging. HY-I intervention attenuated these alterations (Fig. 5C, D). These findings indicate HY-I suppresses SASP-associated inflammation by inhibiting STAT3/NF-κB/NLRP3 signaling and downstream CASP1/IL-18 activation.

### HY-I attenuates renal fibrosis in aged mice

Senescent cells promote extracellular matrix deposition through SASP-mediated chemokines and inflammatory factors, contributing to fibrotic progression in aging kidneys.

**Fig. 6:**
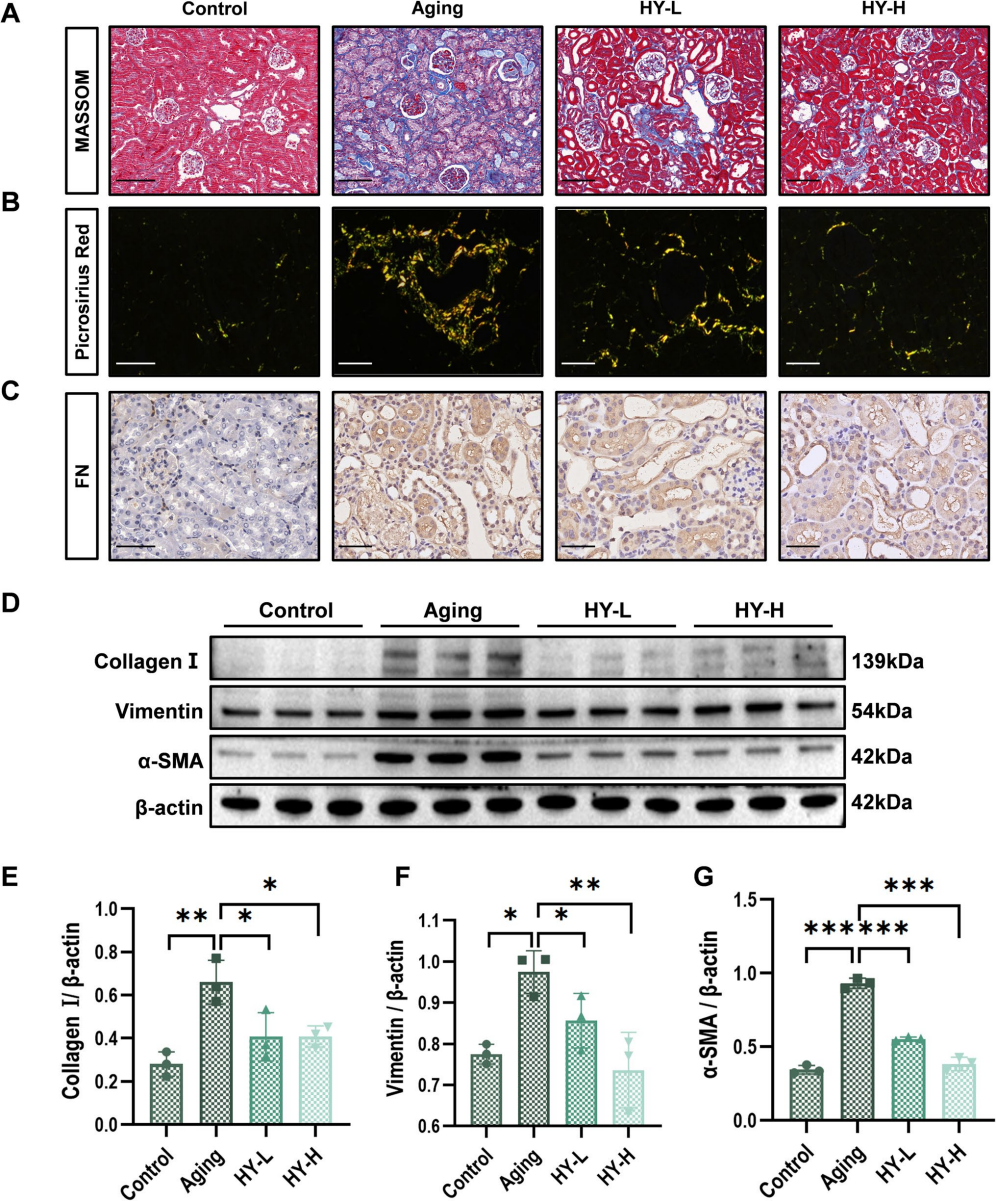
HY-I attenuates renal fibrosis in aged mice. **A**, **B** Masson’s trichrome and Sirius Red staining demonstrating renal collagen deposition. Scale bars, 100 μm. **C** Representative immunohistochemistry of fibronectin (FN) in renal tissues. Scale bars, 50 μm. **D**–**G** Western blot analysis of collagen I, vimentin, and α-SMA expression. (n = 3). One-way ANOVA was used. * indicates comparison with the aging model group; *p < 0.05, **p < 0.01, ***p < 0.001; ns indicates no significant difference

Masson’s trichrome staining revealed reduced collagen deposition in HY-I-treated kidneys (Fig. 6A). Sirius red staining under polarized light demonstrated decreased type I and III collagen accumulation (Fig. 6B). Immunohistochemistry showed attenuated fibronectin diffusion in tubulointerstitial areas (Fig. 6C). Western blotting confirmed downregulation of type I collagen and EMT/fibrosis markers (vimentin, α-SMA) (Fig. 6E).

### Molecular docking analysis of the interaction between HY-I-derived serum components and potential targets

Key serum-absorbed compounds identified through network pharmacology included arjunolic acid, phloretin, 18alpha-glycyrrhetinic acid, pseudolaric acid C, and luteolin.

**Fig. 7:**
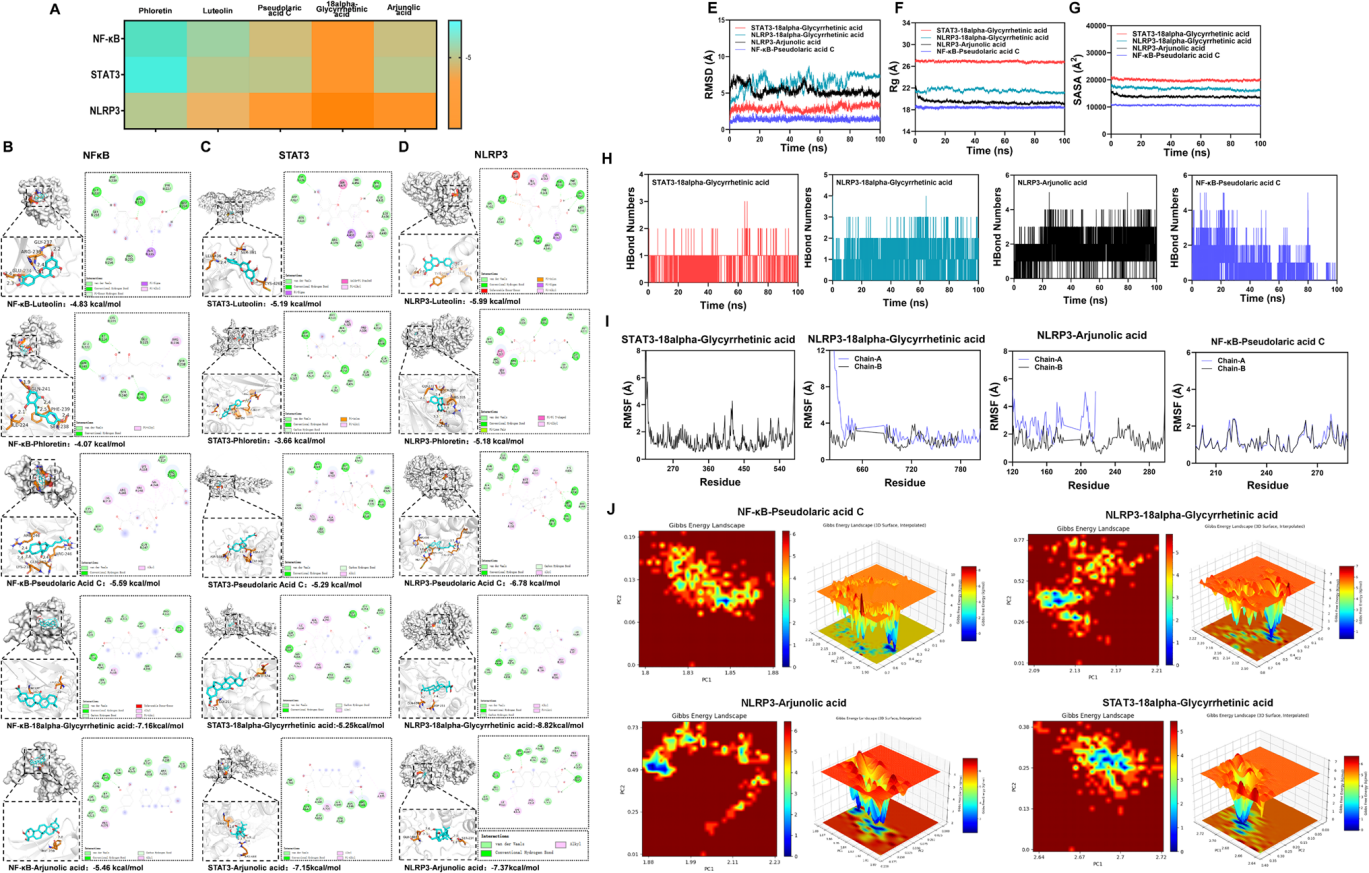
Molecular docking analysis of the interaction between HY-I-derived serum components and potential targets. **A** Binding affinity heatmap of five compounds against three targets. **B** Molecular docking visualization for NF-κB. **C** Molecular docking visualization for STAT3. **D** Molecular docking visualization for NLRP3. **E** Backbone root mean square deviation (RMSD) of four representative ligand-target complexes over 100 ns molecular dynamics (MD) simulation, evaluating the overall conformational stability of the complexes relative to the initial docking structure. **F** Radius of gyration (Rg) of the four complexes during 100 ns MD simulation, assessing the compactness and folding state of the protein tertiary structure. **G** Solvent accessible surface area (SASA) of the four complexes during 100 ns MD simulation, reflecting the solvent exposure level of the protein structure. **H** Time-dependent changes in the number of intermolecular hydrogen bonds between the target protein and its corresponding ligand during 100 ns MD simulation, characterizing the stability of ligand–protein binding interactions. **I** Root mean square fluctuation (RMSF) of each amino acid residue of the target protein in the four complexes, quantifying the flexibility of individual protein residues during the simulation. **J** 2D Gibbs free energy landscape (GEL) and 3D surface visualization of the four complexes, identifying the thermodynamically stable conformation basins of the ligand-bound proteins

Molecular docking using AutoDock demonstrated interactions between these compounds and core targets (NF-κB, STAT3, NLRP3). Among 15 docking configurations (Fig. 7A), multiple exhibited binding energies < -5 kcal/mol, indicating strong binding affinity. Structural visualizations generated in PyMOL (Fig. 7B–D) provide molecular insights into HY-I-mediated suppression of SASP-associated senescence and fibrosis via STAT3/NF-κB/NLRP3 signaling.

We next performed 100 ns all-atom molecular dynamics (MD) simulations on four compound-target complexes with optimal docking affinity (STAT3—18alpha-glycyrrhetinic acid, NLRP3—18alpha-glycyrrhetinic acid, NLRP3-arjunolic acid, NF-κB-pseudolaric acid C) to verify their dynamic binding stability under near-physiological conditions (Fig. 7E–J).

Backbone RMSD analysis (Fig. 7E) confirmed all four complexes reached conformational equilibrium within the simulation period, with no persistent structural drift. The NF-κB-pseudolaric acid C and STAT3—18alpha-glycyrrhetinic acid complexes presented the narrowest fluctuation ranges, indicating superior overall conformational stability.

Consistent with RMSD results, the radius of gyration (Rg, Fig. 7F) and solvent accessible surface area (SASA, Fig. 7G) of all complexes remained stable throughout the simulation, verifying that the target proteins maintained a compact folded state without global unfolding.

Intermolecular hydrogen bond detection (Fig. 7H) showed the NLRP3-arjunolic acid complex maintained the most abundant and continuous hydrogen bond interactions between ligand and protein, while the other three complexes also exhibited sustained hydrogen bond formation as the core driving force for stable ligand anchoring.

RMSF analysis (Fig. 7I) revealed low fluctuation amplitudes of residues within the ligand-binding pocket in all complexes, indicating that ligand binding rigidified the binding region and stabilized the local protein conformation.

Gibbs free energy landscape (GEL) analysis (Fig. 7J) identified well-defined, concentrated low-energy conformational basins for all four complexes, confirming the thermodynamic stability of the ligand-bound states.

Collectively, these MD data validated the stable binding of HY-I’s core active components to their targets at the dynamic molecular level, further supporting the mechanism of HY-I regulating STAT3/NF-κB/NLRP3 signaling to alleviate SASP-related senescence and fibrosis.

## Discussion

The kidneys, responsible for concentrating and excreting metabolites, drugs, and toxins, exhibit high metabolic activity that renders them particularly vulnerable to aging [2]. Structural alterations during renal aging—including tubular atrophy and interstitial expansion—significantly increase susceptibility to CKD and accelerate its progression in the elderly [4]. As primary functional units of the kidney, tubular epithelial cells experience diminished regenerative capacity with advancing age and pathological stress [10]. This accumulation of senescent cells ultimately drives renal functional decline. Critically, senescent cells in aged and CKD kidneys establish a pro-inflammatory, pro-fibrotic microenvironment through SASP secretion [11]. Consequently, targeted modulation of SASP-mediated inflammatory/fibrotic mechanisms represents a crucial therapeutic strategy for age-related CKD. Building on our prior clinical evidence demonstrating HY-I-mediated improvement of renal function in stage 2—3 CKD patients [12], the current study establishes that clinically equivalent HY-I doses ameliorate renal aging and functional deterioration in murine models.

Notably, NF-κB serves as the central transcriptional regulator rapidly activated by senescence stimuli, where it directly controls core SASP components including IL-6 and IL-8 [13]. STAT3 further amplifies and sustains SASP transcription through synergistic cooperation with NF-κB [14]. Downstream of this axis, the NLRP3 inflammasome functions as a critical effector: NF-κB/STAT3 signaling induces NLRP3 and pro-IL-1β transcription, while subsequent NLRP3 activation by damage-associated molecular patterns from senescent cells catalyzes proteolytic maturation of highly inflammatory SASP components IL-1β and IL-18—key mediators that potentiate inflammatory/fibrotic cascades [15]. The STAT3/NF-κB/NLRP3 signaling circuit thus forms a self-reinforcing loop that governs SASP initiation, amplification, and maintenance, making it an optimal therapeutic target for age-related renal decline.

Traditional Chinese Medicine (TCM) demonstrates significant efficacy in modulating aging processes and renal pathologies through the polypharmacological actions inherent to multi-component formulations. Astragalus membranaceus extracts enhance physical function in aging murine models by reversing age-related functional decline [16]. Abelmoschus manihot flavonoids mitigate oxidative stress in D-galactose-induced senescence through reducing reactive oxygen species generation [17]. Centella asiatica contributes triterpenoid compounds such as asiaticoside that exert therapeutic effects across diverse age-related conditions including chronic inflammation, skin photoaging, and neurodegenerative disorders [18, 19], while Salvia miltiorrhiza synergizes with Astragalus to potentiate nephroprotective effects via combinatorial regulation [20, 21]. Glycyrrhiza uralensis serves dual roles as both a harmonizing agent and bioactive source with documented anti-inflammatory and antiviral properties [22, 23]. Cultured Cordyceps mycelia *(Mortierella* sp.)—derived from artificial fermentation of fungal strains isolated from caterpillar larvae—demonstrate established efficacy in clinical renal disease management [24].

In this study, network pharmacology screening identified several key serum-absorbed constituents from HY-I herbal components.

Resveratrol, which has beneficial effects on both inflammation and aging, was selected as a positive control. In our in vivo experiments, high-dose HY‑I intervention showed effects similar to resveratrol in restoring renal function (serum creatinine, uric acid, urinary protein) and reducing the number of senescent kidney cells in aged mice. In terms of improving blood urea nitrogen levels and certain aging molecular markers (Ki67, p16), high-dose HY‑I was superior to resveratrol. Moreover, HE staining revealed that HY‑I was more effective than resveratrol in preserving renal tubular morphology. In our in vitro experiments, HY‑I and resveratrol exhibited comparable effects on improving senescence-associated secretory phenotype (SASP) factors such as inflammatory cytokines; for IL‑18 and other inflammatory factors, HY‑I was slightly more effective than resveratrol alone. These findings suggest that HY‑I not only alleviates aging of the kidney and renal tubular epithelial cells but also has greater potential for improving renal function.

We discovered that HY-I significantly modulates the STAT3/NF-κB/NLRP3-SASP signaling axis, which is critical in renal senescence, with experimental evidence confirming this activity. Molecular docking analysis indicated that five representative compounds derived from the five herbal components of the HY-I formulation interact with STAT3, NF-κB, and NLRP3 through distinct binding modes. For example, Arjunolic acid binding to NF-κB may alter p65 spatial conformation and affect its phosphorylation. Meanwhile, luteolin and other components bind to the pTyr705 pocket in STAT3’s SH2 domain, which could hinder phosphorylation-dependent dimerization and nuclear translocation. NLRP3 assembly may be disrupted by 18α-glycyrrhetinic acid and similar compounds, potentially blocking downstream inflammatory activation. These findings, together with cellular assays and molecular dynamics simulations, suggest a polypharmacological basis for HY-I. This multi-target synergistic mechanism may more effectively block SASP production while conferring additional therapeutic benefits beyond SASP inhibition and improved safety, distinguishing HY-I’s efficacy from single-agent anti-aging drugs such as resveratrol.

HY-I exerts a significant modulatory effect on renal cellular senescence, and these findings provide preliminary mechanistic insights from a multi-target pharmacological perspective. Specifically, HY-I not only effectively suppresses the secretion of senescence-associated secretory phenotype (SASP) factors by renal tubular epithelial cells, but also simultaneously regulates upstream inflammatory triggers. Compared with a single target drug such as resveratrol, which acts on a limited signal pathway, HY-I has advantages in the improvement of some renal function indicators and the protection of renal tubules. Although resveratrol, as a multi‑target inhibitor, provided evidence supporting the inhibitory effect of HY‑I on STAT3/NLRP3, a limitation of this study is the lack of specific STAT3 or NLRP3 inhibitors. Therefore, the specificity of the therapeutic mechanism by which HY‑I targets STAT3/NLRP3 has not been fully established. In summary, the coordinated regulation of the STAT3/NF-κB/NLRP3–SASP axis by HY-I alleviates inflammation and mitigates fibrosis associated with cellular aging, highlighting its potential as a promising therapeutic candidate for the prevention of age-related renal functional decline.

## Conclusion

This study demonstrates that HY-I attenuates renal senescence and fibrosis in both in vivo aging models and in vitro senescence systems by inhibiting the STAT3/NF-κB/NLRP3-SASP axis. These protective effects are mediated through constituent-driven suppression of STAT3 phosphorylation, NF-κB nuclear translocation, and NLRP3 inflammasome assembly. Collectively, our findings provide a mechanistic basis for employing HY-I in clinical management of early-stage CKD to protect against age-related renal deterioration, while advancing its therapeutic potential for further development.

## Data Availability

Data will be made available on request.

## Abbreviations

CKD: Chronic kidney disease
ECM: Extracellular matrix
SASP: Senescence-associated secretory phenotype
ESKD: End⁃stage kidney disease
KA: Kidney aging
HK-2: Human renal tubular cells-2
Res: Resveratrol
CCK-8: Cell counting kit-8
ELISA: Enzyme-linked immunosorbent assay
H&E: Hematoxylin–eosin staining
PAS: Periodic acid-Schiff staining
SA-β-gal: Senescence-associated ß-galactosidase
PPI: Protein–protein interaction
KEGG: Kyoto encyclopedia of genes and genomes
TCMSP: Traditional Chinese medicine systems pharmacology database and analysis platform
D-gal: D-Galactose
Ki67: Marker of proliferation Ki-67
TGFβ1: Transforming growth factor-beta 1
IL-1β: Interleukin-1β
IL6: Interleukin-6
IL18: Interleukin-18
STAT3: Signal transducer and activator of transcription 3
NF-κB: Nuclear factor kappa-B
NLRP3: NOD-like receptor family pyrin domain containing 3
CASP1: Caspase-1
α-SMA: α-Smooth muscle actin
